# Insights into *Acinetobacter baumannii* fatty acid synthesis 3-oxoacyl-ACP reductases

**DOI:** 10.1038/s41598-021-86400-1

**Published:** 2021-03-29

**Authors:** Emily M. Cross, Felise G. Adams, Jack K. Waters, David Aragão, Bart A. Eijkelkamp, Jade K. Forwood

**Affiliations:** 1 School of Biomedical Sciences, Charles Sturt University, Wagga Wagga, NSW 2678 Australia; 2 College of Science and Engineering, Flinders University, Bedford Park, SA 5042 Australia; 3 Australian Synchrotron, Australian Nuclear Science and Technology Organisation, Clayton, VIC 3168 Australia; 4 Diamond Light Source, Harwell Science and Innovation Campus, Didcot, OX11 0DE UK

**Keywords:** X-ray crystallography, Antimicrobial resistance, X-ray crystallography

## Abstract

Treatments for ‘superbug’ infections are the focus for innovative research, as drug resistance threatens human health and medical practices globally. In particular, *Acinetobacter baumannii* (*Ab*) infections are repeatedly reported as difficult to treat due to increasing antibiotic resistance. Therefore, there is increasing need to identify novel targets in the development of different antimicrobials. Of particular interest is fatty acid synthesis, vital for the formation of phospholipids, lipopolysaccharides/lipooligosaccharides, and lipoproteins of Gram-negative envelopes. The bacterial type II fatty acid synthesis (FASII) pathway is an attractive target for the development of inhibitors and is particularly favourable due to the differences from mammalian type I fatty acid synthesis. Discrete enzymes in this pathway include two reductase enzymes: 3-oxoacyl-acyl carrier protein (ACP) reductase (FabG) and enoyl-ACP reductase (FabI). Here, we investigate annotated FabG homologs, finding a low-molecular weight 3-oxoacyl-ACP reductase, as the most likely FASII FabG candidate, and high-molecular weight 3-oxoacyl-ACP reductase (HMwFabG), showing differences in structure and coenzyme preference. To date, this is the second bacterial high-molecular weight FabG structurally characterized, following FabG4 from *Mycobacterium.* We show that Δ*AbHMwfabG* is impaired for growth in nutrient rich media and pellicle formation. We also modelled a third 3-oxoacyl-ACP reductase, which we annotated as *Ab*SDR. Despite containing residues for catalysis and the ACP coordinating motif, biochemical analyses showed limited activity against an acetoacetyl-CoA substrate in vitro. Inhibitors designed to target FabG proteins and thus prevent fatty acid synthesis may provide a platform for use against multidrug-resistant pathogens including *A. baumannii*.

## Introduction

Antibacterial resistance is a large burden to modern healthcare, and is predicted to result in 10 million deaths annually if no action is taken by 2050^49^. In particular, *Acinetobacter baumannii*, a Gram-negative opportunistic pathogen, is highlighted as a major concern to human health. *A. baumannii* is implicated in both nosocomial and community acquired infections^24^, and its pathogenic success can be largely attributed to its highly plastic genome, resulting in the acquisition of numerous virulence and resistance determinants enabling it to thrive in hospital environments^31,50,69^. Specifically, its ability to form biofilms on medical equipment contributes to severe nosocomial infections^31^. Increasing drug resistance has resulted in the use of last resort antibiotics, including tigecycline and colistin, and pan-drug resistant strains may become common within the next two decades^70^. Carbapenem-resistant *A. baumannii* was recently listed as an urgent threat by the Centre for Disease Control (CDC) and as the top priority for drug development by the World Health Organisation (WHO)^12,62^. For these reasons, research into novel drug development is essential for successful treatment of multi-drug resistant pathogens, including *A. baumannii*.

Fatty acids can be produced via two distinct pathways: type I and type II Fatty Acid Synthesis (FASI and FASII, respectively). In mammals, FASI is performed by an enzyme with a large multidomain and multifunctional structure^6^. In contrast, bacteria utilise the FASII system which involves a series of discrete and functionally specific enzymes that produce fatty acids primarily destined for incorporation into membrane lipids. Acyl-carrier protein (ACP) is attached to the acyl chain via a thioester linkage and acts to deliver the substrate between enzymes of the FASII pathway. In brief, this pathway begins with condensation of acetyl-CoA and malonyl-ACP by the Fatty acid biosynthesis (Fab) enzyme, FabH. This is followed by reduction of the β-keto group by FabG, dehydration of the substrate by FabZ or FabA, and then reduction by FabI, FabL, FabK, or FabV. Condensation by FabB or FabF begins a new cycle which repeats, elongating the fatty acid chain by two carbons each cycle. Due to the differences between FASI and FASII, drugs designed to target non-homologous bacterial enzymes are attractive in antimicrobial development^11,66^. FabG (3-oxoacyl-ACP or β-ketoacyl-ACP reductase) converts β-ketoacyl-ACP to β-hydroxyacyl-ACP in the presence of NADH or NADPH. Complicating drug development, bacteria can harbour multiple FabI isoforms (eg FabI/FabL/FabK/FabV), however *A. baumannii* contains only a single FabI^39^. Similarly, prokaryotes can also contain multiple FabG isoforms, as exemplified in *Mycobacterium.* These are designated as FabG1/MabA (Rv1483), FabG2 (Rv1350), SDR (Rv2002), FabG4 (Rv0242c), and FabG5 (Rv2766c)^14^. FabG1 is a classical NADPH dependent short chain dehydrogenase reductase (SDR), alternatively, FabG4 of *Mycobacterium tuberculosis* (*Mt*FabG4) is a much larger protein with preference for NADH rather than NADPH, designated as a high molecular weight (HMw) FabG^13,19^. FabG enzymes belong to the vast superfamily of SDRs that play a broad number of biological roles in eukaryotes, prokaryotes, and archaea, typically sharing 15–30% sequence identity and ‘fingerprint’ motifs^37^. Because SDR proteins show such a diverse range of functions whilst maintaining structural features, it can be difficult to assign biological function from sequence or structural information alone. Structures for classical and extended SDRs from *A. baumannii* have been reported previously^16,55,56^.

The development of antimicrobials targeting FASII processes is validated, and current drug examples include triclosan and isoniazid (targeting the enoyl reductase enzyme, FabI), and cerulenin (targeting the condensing enzymes FabB and FabF)^11,66,72^. Currently, only FabI (catalysing the second reductase of FASII) has been structurally elucidated from the FASII pathway of *A. baumannii,* also in complex with a promising FabI inhibitor, AFN-1252^41,53,54^. Limited research has investigated FabG inhibitors against bacteria, however, some natural products, including EGCG/plant polyphenols and Macrolactin S isolated from *Bacillus*, have been studied^59,75^. It may also be proposed that due to similar structure and function of FabG to the second reductase, FabI, drugs could promote dual inhibition, however, this remains to be confirmed experimentally.

The current study investigates potential candidates for the role of the FASII reductase enzyme FabG in *A. baumannii*. We report the structure and activity of what we suggest is the primary FabG (ABO11256.2), with preference for NADPH. We also reveal *Ab*SDR (ABO10977.2), a FabG-like protein with limited activity towards acetoacetyl-CoA in vitro. Finally, we report a HMwFabG (ABO12488.2) in *A. baumannii*, with a dimeric structure, preference to NADH, and similarities to *Mt*FabG4. Deletion of the *HMwFabG* from *A. baumannii* ATCC 17978 resulted in impaired growth in nutrient rich media and a compromised ability to form a pellicle.

## Methods and materials

### Genome mining and phylogenetic analysis

Identification of FabG-like protein sequences across a panel of *A. baumannii* genomes from different clonal groups (*A. baumannii* AB5075_UW, AYE, WM99c, ACICU, ATCC 17978, D1279979 and SDF) were performed using V5VHN7 and A0A0E1FTA3 (UniProtKB) as queries for BlastP searches against the NCBI non-redundant protein sequence database^4^. Duplicate sequences obtained from independent BlastP searches were removed. All FASTA sequences (n = 175) were used as input data and a multiple sequence alignment performed using ClustalW as an integrated part of GenomeNet^36^ with default settings applied. Phylogenetic reconstructions from the multiple sequence alignment were performed using the “build” function of ETE3 v3.1.1 in GenomeNet. Protein phylogeny was examined using the midpoint rooted Maximum-Likelihood method (100 bootstraps) using RAxML v8.1.20 with model PROTGAMMAJTT and default parameters applied^60^. The resulting tree was annotated in FigTree v1.4.4 (http://tree.bio.ed.ac.uk/software/figtree/). All *A. baumannii* genomes included in the phylogenetic analyses were aligned using Mauve^17^ to confirm the presence/absence of identified SDRs and their relative genetic positioning within the examined genomes. Identification of conserved SDR fingerprint motifs from FASTA sequences were performed manually. To determine ACP interacting residues from the identified *A. baumannii* SDRs, representative protein sequences ([ST]x_12_Yx_3_K) from clades with fully conserved active site motifs (n = 28) were aligned against known FabG sequences using methods as described above with gap open penalties set at 12.

GenBank accession numbers used in protein phylogenetic analyses: *A. baumannii* AB5075_UW, CP008706; *A. baumannii* AYE, NC_010410; *A. baumannii* WM99c, AERY00000000; *A. baumannii* ACICU, CP000863; *A. baumannii* ATCC 17978, CP000521; *A. baumannii* D1279979, AERZ00000000; *A. baumannii* SDF, CU468230.

### Expression and purification

Genes encoding the putative enzymes were codon optimized for *Escherichia coli* expression and cloned into the pMCSG21 vector at the *Ssp*I site, producing a 6-His fusion protein with TEV cleavage site for tag removal (Genscript, Piscataway, NJ). Plasmids were transformed into competent *E. coli BL21(DE3) pLysS* cells (Merck-Millipore) and selected on spectinomycin (100 µg/mL) Luria–Bertani (LB) agar plates. A starter culture was used to inoculate 1 L of auto-induction media^61^, incubated at room temperature for 36 h until an OD_600_ > 3.0 was reached. Cells were harvested by centrifugation and resuspended in ‘Buffer A’ containing 20 mM imidazole, 300 mM NaCl, 50 mM phosphate buffer pH 8.0. Cell membranes were lysed via two freeze–thaw cycles followed by the addition of 2 mg/mL lysozyme and 0.025 mg/mL DNAse. The cell lysate was clarified using centrifugation and filtration (using a 0.45 μm PVDF syringe filter) prior to purification over a 5 mL Nickel-Sepharose HisTrap HP column (GE Healthcare). A 10-column volume wash with Buffer A removed unbound contaminants. Following this, the sample was eluted by a gradient of elution 'Buffer B' (containing 50 mM phosphate buffer pH 8.0, 300 mM NaCl, and 500 mM imidazole) over 5-column volumes. The resulting eluate was treated with TEV protease overnight to cleave the affinity tag, then further purified using a Superdex 200 26/60 column (GE Healthcare) in tris-buffered saline (50 mM Tris pH 8.0, 125 mM NaCl). The homogenous peak from gel filtration was collected and concentrated using a 10 kDa MW centrifugal filter (Amicon/Millipore) and all samples were assessed for purity by SDS-PAGE.

### Crystallization, data collection, and structure modelling

Purified protein samples were trialled against a number of commercial screens using hanging-drop vapour diffusion in 48-well plates. Above 300 μL of reservoir solution, a siliconized glass coverslip held a hanging-drop consisting of 1.5 μL of concentrated protein mixed with 1.5 μL of reservoir condition. FabG crystals formed in the condition containing 0.1 M Tris pH 8.0 and 2 M ammonium sulphate. Crystallization of FabG with NADPH was performed with a 1:10 molar ratio of protein to coenzyme in the same reservoir solution as the apo crystals. HMwFabG crystals formed in 0.1 M HEPES sodium pH 7.5, 10% v/v 2-Propanol, 20% w/v PEG 4000 (crystal form 1) and 0.2 M sodium malonate pH 5.0 and 20% w/v PEG 3350 (crystal form 2). *Ab*SDR crystals formed in 0.1 M magnesium acetate, 0.1 M sodium acetate pH 4.5, and 8% w/v PEG 8000. All crystals were cryo-protected with 20% v/v glycerol and flash cooled with liquid nitrogen. Diffraction data were collected at the Australian Synchrotron using the MX1 and MX2 macromolecular beamlines^5,15^. Data was analysed using iMosflm^9^, Aimless^23^, Phaser^47^, Phenix^2^, and Coot^22^. PDBsum was used to investigate the macromolecular structures and interactions^42^. PDB depositions are annotated under the codes 6UDS, 6WPR, 6UUV, 6UUT, and 6NRP.

### Enzyme assays

Enzyme assays were performed using previously established methods^16,48^. Briefly, these assays involved monitoring a decrease in absorbance at 340 nm indicating oxidation of NADH or NADPH. Each assay contained 1 µg of enzyme, 500 µM acetoacetyl-CoA, 200 µM coenzyme (NADH or NADPH), and assay buffer (20 mM HEPES pH 7.4, 125 mM NaCl) to a total volume of 100 µL. Absorbance readings were taken using an Epoch microplate reader (Biotek), in triplicates before and after the addition of acetoacetyl-CoA with 30 min incubation at 37 °C.

### Bacterial strains, media, and growth conditions

*A. baumannii* ATCC 17978 was obtained from the American Type Culture Collection (ATCC). ATCC 17978 and Δ*AbHMwfabG* were routinely cultured using LB broth or LB agar plates (1.5%) and incubated under aerobic conditions at 37 °C unless otherwise stated. The *A. baumannii* ATCC 17978 ∆*AbHMwfabG* generated as part of this study using an established strategy^1,64^. (See Supplementary Tables 1 and 2 for list of strains and oligonucleotides used in the study, respectively).

**Table 1:** Data-collection and model statistics. Overall and (outer shell).

	FabG apo	FabG NADPH	HMwFabG CF1	HMwFabG CF2	*Ab*SDR
PDB Code	6UDS	6WPR	6UUV	6UUT	6NRP
Resolution range (Å)	29.13–1.90 (1.94–1.90)	50.55–1.85 (1.89–1.85)	48.71–1.80 (1.84–1.80)	80.32–1.65 (1.68–1.65)	24.99–1.90 (1.93–1.90)
Space group	P 4_3_ 2_1_ 2	P 4_3_ 2_1_ 2	P 1	P 1 2_1_ 1	P 3_1_ 2 1
Unit cell length (Å)	a = 87.39, b = 87.39, c = 151.29	a = 87.43, b = 87.43, c = 151.65	a = 59.19 b = 63.65 c = 64.36	a = 64.60 b = 80.32 c = 76.58	a = 89.48, b = 89.48, c = 239.47
Unit cell angle ( ° )	α = 90.00 β = 90.00 γ = 90.00	α = 90.00 β = 90.00 γ = 90.00	α = 69.70 β = 65.45 γ = 76.63	α = 90.00 β = 100.38 γ = 90.00	α = 90.00, β = 90.00, γ = 120.00
Total observations	841,265 (47,674)	729,944 (44,809)	242,995 (12,912)	298,276 (15,000)	770,871 (32,908)
Unique reflections	46,833 (3036)	51,013 (3085)	71,417 (4191)	92,484 (4543)	88,460 (4426)
Multiplicity	18.0 (15.7)	14.3 (14.5)	3.4 (3.1)	3.2 (3.3)	8.7 (7.4)
Completeness (%)	99.8 (98.2)	100 (100)	96.6 (95.6)	99.8 (99.7)	99.9 (99.5)
Mean I/Sigma (I)	9.6 (2.0)	26.3 (4.9)	9.5 (2.7)	8.2 (3.6)	9.1 (2.9)
CC (1/2)	0.997 (0.781)	1.000 (0.939)	0.996 (0.809)	0.995 (0.872)	0.996 (0.637)
R-pim	0.040 (0.288)	0.021 (0.158)	0.044 (0.273)	0.050 (0.191)	0.050 (0.236)
R-meas	0.169 (1.166)	0.080 (0.606)	0.082 (0.487)	0.093 (0.354)	0.148 (0.652)
Wilson B Factor	28.803	14.109	16.660	12.232	15.161
R-work	0.1881 (0.2845)	0.1603 (0.2044)	0.1814 (0.2527)	0.2018 (0.2311)	0.1702 (0.2069)
R-free	0.2064 (0.3353)	0.1905 (0.2733)	0.2145 (0.2876)	0.2350 (0.2633)	0.1917 (0.2473)
Number of non-hydrogen atoms	3869	4191	6677	7013	7616
RMS (bonds) (Å)	0.005	0.010	0.008	0.007	0.008
RMS (angles) (°)	0.80	1.07	1.16	1.15	1.20
Ramachandran favored (%)	97.08	97.46	97.71	98.08	96.54
Ramachandran allowed (%)	2.92	2.54	2.29	1.92	3.46
Ramachandran outliers (%)	0.00	0.00	0.00	0.00	0.00
Rotamer Outliers (%)	0.00	0.53	0.00	0.00	0.39
Clashscore	0.85	0.68	2.01	1.67	2.77
Average B-factor	39.07	22.53	27.19	17.48	22.13

### Growth in nutrient rich and nutrient poor conditions

ATCC 17978 and Δ*AbHMwfabG* were grown overnight (ON) in LB broth, sub-cultured to an optical density at 600 nm (OD_600_) of 0.01 in LB. For growth in nutrient poor conditions, cells were pelleted and washed twice with phosphate buffered saline (PBS), and resuspended in 1 mL of M9 minimal media and sub-cultured to an OD_600_ of 0.05 in M9 minimal media. M9 minimal medium was made using a stock solution of 10X M9 salts (400 mM Na_2_HPO_4_.7H_2_O, 220 mM KH_2_PO_4_, 86 mM NaCl and 186 mM NH_4_Cl) and subsequently diluted 1:10 and supplemented with 2 mM MgSO_4_ and 0.1 mM CaCl_2_. Cells (200 µl) were transferred to 96-well microtitre plates (GreinerBio-one), sealed with parafilm and kept in a humidity box at 37 °C ON under static conditions. To determine growth, cells were resuspended and measured at OD_600_ on a Spectrostar nano (BMG Labtech).

### Pellicle and biofilm formation analyses

ATCC 17978 and *ΔAbHMwfabG* were grown ON in LB broth, sub-cultured to OD_600_ of 0.05 in LB broth (5 mL) in polypropylene tubes and grown in the dark for 72 h at 37 °C under static conditions. Quantitative measurement of pellicles was assessed by the addition of 1 mL of methanol, the floating pellicle was subsequently removed and resuspended in 1 mL of PBS. Resuspended pellicles and planktonic growing bacteria (200 µL) were transferred to 96-well microtitre plates (GreinerBio-one) and OD_600_ values determined using a Spectrostar nano (BMG Labtech). Following removal of the remaining culture media, biofilm was quantified by crystal violet staining, thorough washing and quantitation by measuring the optical density at 590 nm using previously described methods^21^.

### Motility

Motility analyses of strains ATCC 17978 and Δ*AbHMwfabG* were performed by inoculating colony material in the centre of a semi-solid LB agar plate (0.35% Eiken Agar) as per previous studies^3^. Bacterial migration was quantified (in centimetres) 10 h post inoculation at 37 °C.

### Minimal inhibitory concentration analyses

The minimal inhibitory concentration (MIC) of strains ATCC 17978 and ∆*AbHMwfabG* was determined using a previously described method^68^. In brief, overnight cultures were diluted in cation-adjusted Mueller–Hinton media. Cells were then transferred to a 96-well microtiter tray containing a two-fold dilution series of colistin, chloramphenicol, gentamicin or streptomycin, with the final volume being 100 μL. Plates were incubated in a humidified chamber at 37 °C for 18 h. Growth was determined by visual examination.

## Results and discussion

### In silico identification of *A. baumannii* SDRs

Given numerous open reading frames in *A. baumannii* have been annotated as putative 3-oxoacyl-ACP reductases (i.e. FabG), in silico analyses were performed to delineate the FabG homolog responsible for catalysing the reduction of β-ketoacyl-ACP substrates in the FASII pathway from other SDRs. Protein sequences that shared significant identity to distinct SDR members from seven distinct *A. baumannii* strains were comprehensively examined.

Two isolates from the international clone (IC) I lineage (AB5075_UW and AYE) and two from the IC II lineage (ACICU and WM99c) were included. The remaining three isolates examined are not categorized within the described IC lineages. Two of these are the widely studied strains ATCC 17978 and SDF, where the latter has undergone significant insertion sequence (IS) mediated genome reduction and is defined as avirulent^27^. The *A. baumannii* strain D1279779 was isolated from an outpatient in Northern Australia and represents a community-acquired *A. baumannii* isolate^24^.

**Figure 1:**
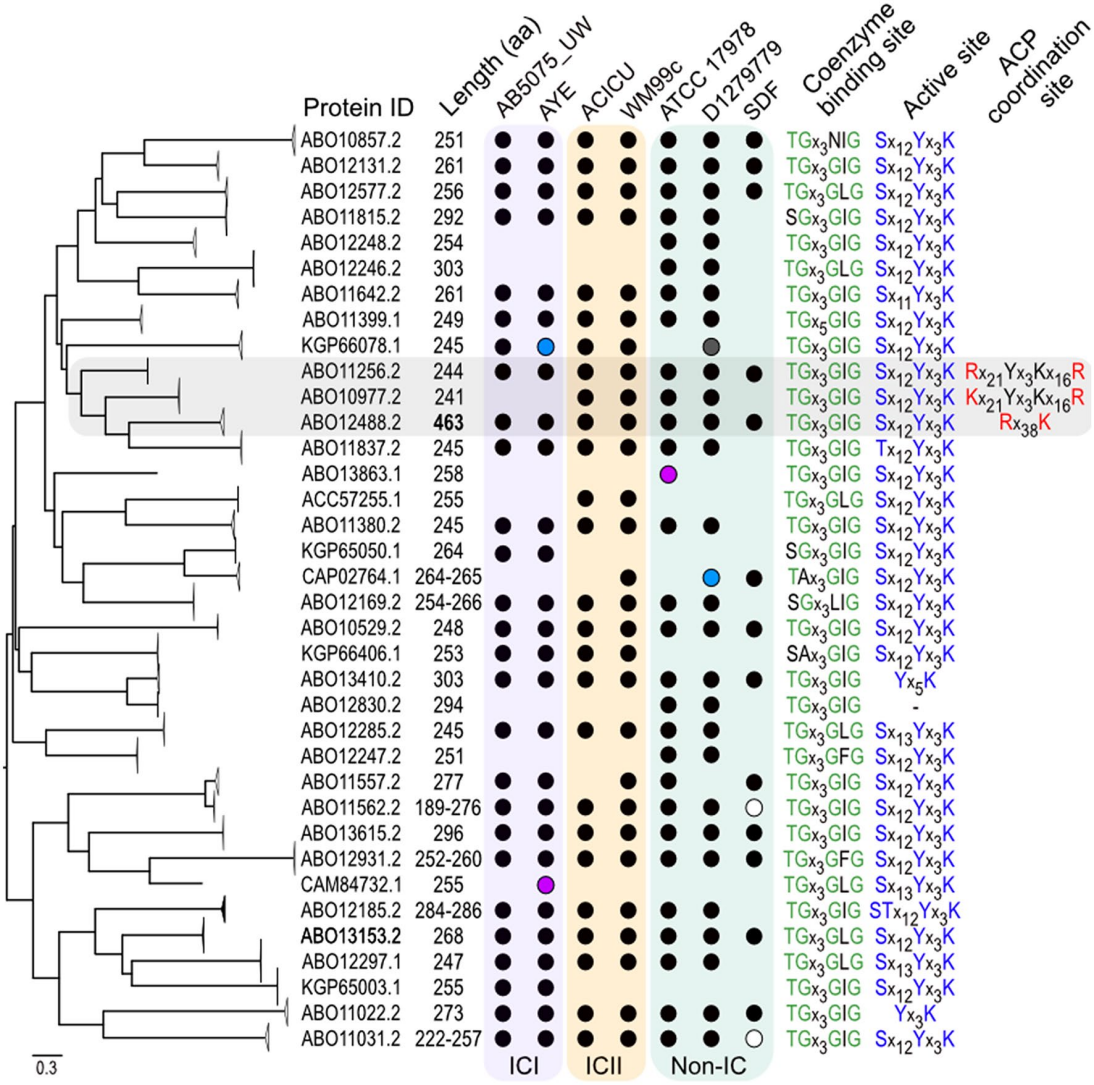
Bioinformatic analysis of the *A. baumannii* short-chain dehydrogenase/reductases. *A. baumannii* short-chain dehydrogenase/reductases are separated into multiple distinct phylogenetic clades. A midpoint rooted maximum likelihood phylogenetic tree was generated from 175 aligned putatively annotated SDR protein sequences identified from seven distinct *A. baumannii* strains; AB5075_UW, AYE, ACICU, WM99c, ATCC 17978, D1279779 and SDF. Clades were collapsed for clarity in FigTree v1.4.4 and subsequently labelled using a representative protein accession ID. The consensus protein length identified for each clade are displayed in amino acids (aa). Bold-face type represents a putative high molecular weight 3-oxoacyl ACP reductase. Black filled circles represent the presence of a protein sequence within a given clade from the investigated *A. baumannii* genomes. Blue and white filled circles indicate protein sequences that are divided across two open reading frames and have C-terminal truncations, respectively. Purple filled circles indicate sequences present on endogenous plasmids. Grey filled circle indicates the presence of a sequence but encoded at a different genetic location to other identified clade members. Transparent purple, orange and green boxes indicate strains classified as international clone I (ICI), international clone II (ICII) and non-international clone (Non-IC) lineages, respectively. Consensus sequences of coenzyme binding and catalytic active site fingerprint motifs from each clade are shown, with conserved residues coloured in green and blue font, respectively. Consensus sequences of conserved charged residues responsible for coordination of acyl-acyl carrier protein (ACP) binding are shown in red font. See Supplementary Figure 1 for comparative alignment of short-chain dehydrogenase/reductases. ACP-coordinating residues for the putative high molecular weight FabG of *A. baumannii* (clade ABO12488.2) were identified using a comparative alignment against FabG4 of *Mycobacterium tuberculosis*^19^. Transparent grey box indicates clades with potential 3-oxoacyl ACP reductase activity. *A. baumannii* protein identifiers are as follows; ATCC 17978, (ABO); AB5075_UW, (KGP); ACICU, (ACC); SDF, (CAP) and AYE, (CAM).

A total of 175 protein sequences were identified, where phylogenetic analysis revealed clustering of these sequences into 36 distinct clades (Fig. 1). The length of proteins identified from 35 of the 36 clades ranged between 241 and 303 amino acids, indicative of members belonging to the classical family of SDRs^38^. In contrast, all members of the clade represented by ABO12488.2 were 463 amino acids in length.

The majority of SDRs were present in two or more strains, with two exceptions identified (ABO13863.1 and CAM84732.1). These members were found to be encoded on endogenous plasmids present in ATCC 17978 and AYE, respectively, and thus suggest a foreign origin. The non-IC lineage strains ATCC 17978 and D1279979 encoded the greatest number of distinct SDR proteins (n = 29) from the examined *A. baumannii* genomes. The higher abundance can be directly attributed to unique SDR sequences being encoded within ‘regions of genomic plasticity’ unique to these strains, as previously defined by Farrugia and colleagues^24^. Clades represented by KGP65003.1 and KGP65050.1 were found to be specific for the IC I lineage, as they were only present in AYE and AB5075_UW strains whilst the clade represented by ACC57255.1 was the only IC II specific SDR identified. The avirulent *A. baumannii* strain SDF was found to encode 15 distinct SDRs where two sequences encoded C-terminal truncations as a result of IS*Aba7* mediated insertions. It could be hypothesised that sequences conserved within SDF catalyze essential functions whilst homologs absent from this strain may indicate redundancy or catalyze functions that enable metabolic versatility and/or virulence. Our analyses indicate significant diversity of SDRs present in *A. baumannii*, where the presence of specific members/proteins are influenced by the evolution of distinct global lineages and the impressive genetic plasticity afforded by this bacterium.

### Comparative analysis of conserved SDR domains

All identified sequences were found to harbour the N-terminal coenzyme binding fingerprint motif most similar to the consensus of the classical SDR family, TG_X3_[AG][FILV]G^38^. Members of five distinct clades were found to encode amino acid substitutions at important conserved residues within the motif, potentially indicating reduced or no functional activity.

Twenty-nine of the 36 clades harboured the classical SDR catalytic triad active site consensus sequence, [ST]x_12_Yx_3_K^38^. No residues constituting the active site sequence could be identified for the two clade members as represented by ABO12830.2 and thus are likely non-functional. The remainder of sequences that differed from the consensus were due to deviation from the consensus sequence pattern or absence of the serine/threonine residue critical for substrate stability.

### Identification of *A. baumannii* FabG homologs with putative 3-oxoacyl ACP functionality

Similar to many FASII enzymes, FabG homologs encode a conserved ACP-binding signature surface which facilitates ACP docking and subsequent donation of the acyl chain from the ACP prosthetic group^76^. It has been shown that Arg_129_ and Arg_172_, residues located at the entrance to the active site tunnel play a significant role in ACP docking in *E. coli*^76^. FabG is deemed essential in most bacteria as it is the only isozyme capable of reducing β-ketoacyl-ACP substrates required for endogenous fatty acid production, however, some unique exceptions have been reported^25,35,45,73^. To identify SDRs with putative 3-oxoacyl ACP activity in *A. baumannii*, representative protein sequences from distinct clades encoding the typical catalytic motif ([ST]x_12_Yx_3_K) were aligned against sequences with known reductive capacity towards 3-oxoacyl ACP substrates (Supplementary Figure 1). The multiple sequence alignment revealed that sequences from clades represented by ABO11256.2 and ABO10977.2 encoded the conserved hydrophobic residues adjacent to the active site which support ACP interaction. ABO11256.2 is conserved across all examined genomes and is encoded in an operon with other enzymes with predicted roles in fatty acid biosynthesis. Further, large scale mutagenesis studies have defined ABO11256.2 to be the only SDR that is essential for *A. baumannii* viability, and thus is likely to be the primary FASII FabG of the bacterium^28,65^. Interestingly, ABO10977.2 is only present in four of the seven genomes, where it was absent from the IC I isolates AYE and AB5075_UW as well as SDF (Fig. 1). These phylogenetic analyses have deduced clades with ACP interacting residues to be genetically similar, and form sister clades with ABO12488.2, a conserved clade where all members are approximately double in length to that of ABO11256.2 and ABO10977.2.

To further explore the unique SDR, the representative ABO12488.2 sequence was used as a query for conserved domain searches which revealed that members of this clade harbor an N-terminal flavodoxin-type domain and a typical ketoreductase domain at its C-terminus, indicative of HMwFabG proteins such as *Mt*FabG4^19^. HMwFabG represent a genetically distinct group of β-oxoacyl reductases with homologs often restricted to species from Actinobacteria and Proteobacteria phyla and catalyze the reduction of β-oxoacyl-ACP using NADH as a coenzyme^19^. Similar to low molecular weight FabG proteins, hydrophobic residues are also responsible for interactions with ACP substrates albeit in a different position (Arg_111_ and Lys_150_)^20^. A multiple sequence alignment of *Mt*FabG4 against ABO12488.2 clade members revealed the conservation of Arg_111_ and Lys_150_ (Arg127 and Lys166 in ABO12488.2) residues, inferring the HMwFabG member of *A. baumannii* can also catalyze the reduction of β-oxoacyl-ACP substrates.

By using comparative analyses, we have deduced *A. baumannii* SDR members with putative 3-oxoacyl ACP activity.

### Structural analysis of *A. baumannii* FabG; a primary low molecular weight FabG

**Figure 2:**
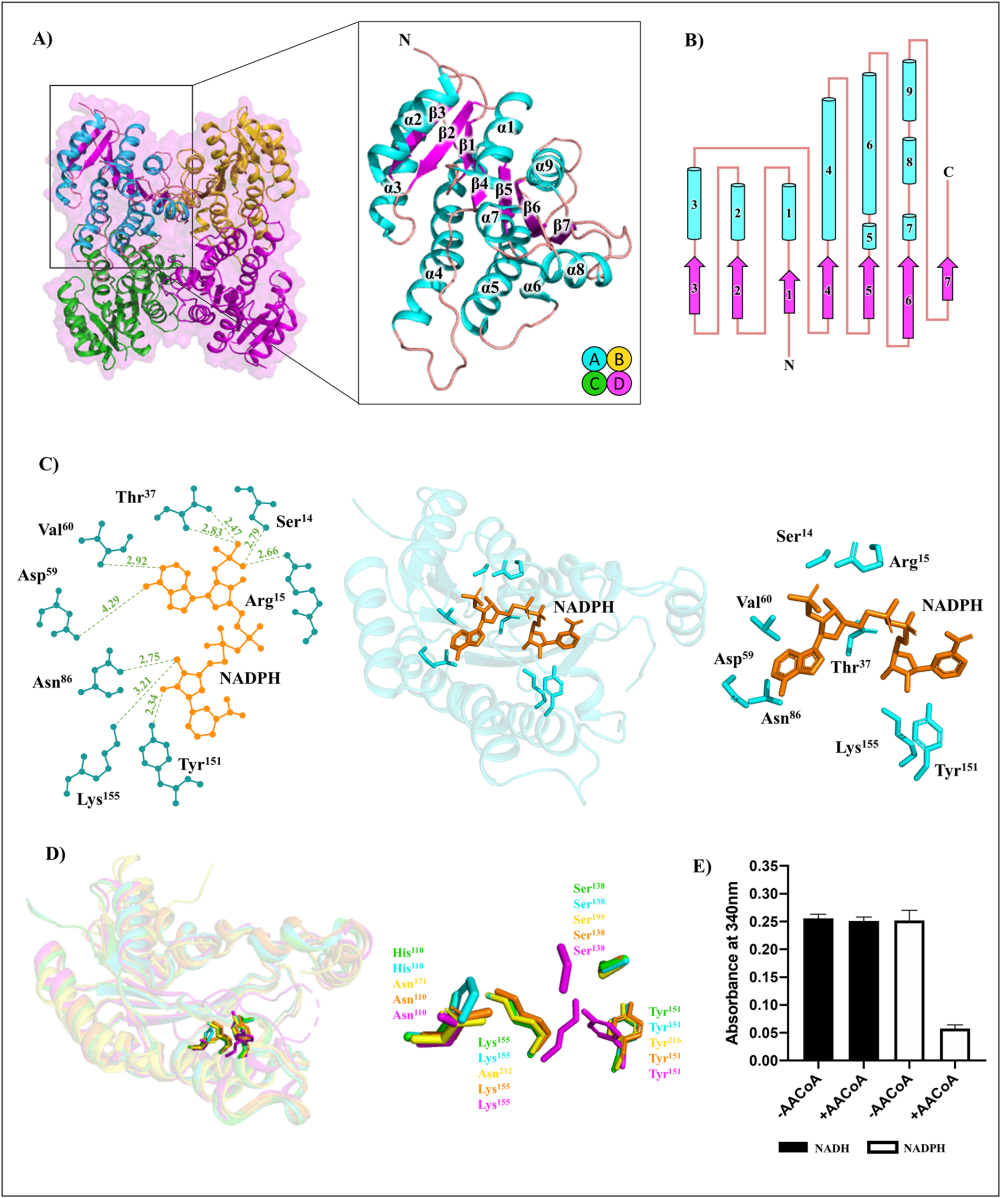
Structure of *A. baumannii* FabG (ABO11256.2) (**A**) The quaternary assembly and protomer displaying a Rossmann fold within the secondary structure. This model was deposited in the PDB under the code 6UDS. (**B**) Topology diagram of protomer assembly with loops (salmon), α-helices (cyan), and β-sheets (magenta). (**C**) Structure of NADPH (orange) bound FabG. The coenzyme is positioned by several hydrogen bonds (green, distances in Å) between NADPH and surrounding residues (cyan). The FabG:NADPH model was deposited in the PDB under the code 6WPR. (**D**) Comparison of FabG active site residues in active and inactive conformations. *E. coli* FabG catalytic residues show conformational changes between the NADP bound ‘active’ (1Q7B/A, orange) and apo ‘inactive’ (1I01/B, magenta) proteins^51,52^. Both apo (PDB: 6UDS/A, green) and NADPH (PDB: 6WPR/A, cyan) structures of *A. baumannii* FabG show the same ‘active’ residue architecture regardless of coenzyme. Similarly, apo FabG from *P. falciparum* shows an active arrangement (2C07/A, yellow)^67^. (**E**) FabG enzyme activity assay using acetoacetyl-CoA substrate. All figure images were generated/drawn by the authors.

Our in silico data has elucidated that FabG (ABO11256.2) is conserved across *A. baumannii* strains and has been previously shown to be essential for cell viability^28^. We therefore pursued techniques in X-ray crystallography to elucidate the three-dimensional structure of this important enzyme. The structure of apo FabG was resolved at 1.9 Å in the space group P 4_3_ 2_1_ 2 using molecular replacement with starting model PDB: 4WJZ (58% sequence identity, Hou et al.^34^). The structural model displayed good stereochemistry, with a final overall R_work_ = 0.19 and R_free_ = 0.21 (complete statistics in Table 1). The model showed two protomers in the crystal asymmetric unit with each protomer consisting of 244 residues arranged as an archetypal Rossmann fold (Fig. 2A). The secondary structure displayed a central β-sheet (β3-β2-β1-β4-β5-β6-β7) skirted by 9 α-helices (Fig. 2B). An α7 turn α8 motif forms a capping region, or flexible ‘lid’ to the active site cavity. This capping region is observed in most SDRs, and its flexibility is reflected with poorer density and higher B factors in this region. Overall, from generation of crystal symmetry mates, the tertiary structure is a homotetramer arranged as two anti-parallel dimers, consistent with conventional FabG enzymes, and the elution profile on a size exclusion column (≈ 100 kDa, or 4 × 26 kDa protomers). Interactions between chains were confirmed by PDBsum (Supplementary Table 3)^42^. A/B interactions involve an average total interface area of 1502 Å^2^, and 12 hydrogen bonds at the β7 strand of each opposing protomer. The interaction between protomers B and C is made through the α4 to α4′, α5′ to α5, and α5- β5 loop regions of each chain with an average total interface area of 1492.5 Å^2^, 6 salt bridges, and 22 hydrogen bonds. No interactions were observed between diagonal protomers, A/D and B/C.

In terms of catalytic mechanism, the reduction reaction is well understood and occurs when a hydride from the coenzyme nicotinamide ring and a proton from an active site tyrosine is donated to the C3 carbon and C3 oxygen of the substrate β-ketoacyl-ACP, respectively^26,52^. For SDR proteins, the catalytic residues are highly conserved. In FabG this triad is well positioned to accept the hydride ion and includes Ser_138_, Tyr_151_ located on the loop region between β5 and α7, and Lys_155_ located on the α5 helix. Following the reaction, it is known that a proton relay system replenishes the tyrosine via a residue (commonly Asn, Gly, or Ser; position Asn_110_ in *E. coli*) that forms a kink in the α4 helix near the active site, crucial for positioning a backbone carbonyl for participation in a water network^26,52^. Whilst an asparagine residue is commonly observed at this site, our FabG shows a His_110_ residue in this location, however, it is the kink and the helical backbone that is essential rather than the residue sidechain, and this kink is observed in α4 of our structure.

Specific residues that determine coenzyme preference in SDR proteins has been elucidated and coenzyme binding families assigned^38,63^. For FabG, the presence of a basic residue in the Gly motif (TGAS**R**GIG_18_) suggested preference for NADPH and categorization into the cP1 subfamily^38^. To observe binding mechanisms, we performed co-crystallization with NADPH. Crystals diffracted on the MX1 beamline at the Australian Synchrotron to a resolution of 1.85 Å in the spacegroup P 4_3_ 2_1_ 2 and the final model displayed good stereochemistry and an overall R_work_ and R_free_ of 0.16 and 0.19 respectively (Table 1). The crystallographic asymmetric unit revealed two protomers, and strong positive density allowing NADPH to be modelled within each protomer. The NADPH is bound through an extensive binding pocket, whereby the nicotinamide moiety is bonded with Lys_155_ and Tyr_151_, and the adenine ribose is bonded through Ser_14_, Arg_15_, Thr_37_, Asp_59_, Val_60_, and Asn_86_ (Fig. 2C). Interestingly, contrary to previous reports of large conformation changes in FabG upon coenzyme binding (such as that in *E. coli*), our structure reveals little change at the active site between apo and holo proteins (chain A comparison reveals r.m.s.d = 0.107 Å)^51,52^. An ‘active’ arrangement of catalytic residues for apo proteins is also observed in *Plasmodium falciparum* PDB: 2C07^67^, *Staphylococcus aureus* PDB: 3OSU (*unpublished)*, and *Bacillus anthracis* PDB: 2UVD^74^. Superimposition of *A. baumannii* FabG ± coenzyme, *E. coli* FabG ± coenzyme, and *P. falciparum* apo FabG highlight the positioning of catalytic residues at the active site in these models (Fig. 2D). Enzyme assays complemented clear preference for NADPH by FabG (Fig. 2E), confirming biological relevance for the NADPH bound structure. Although enzyme assays were conducted in the presence of a substrate mimic, acetoacetyl-CoA, we believe FabG has an ability to bind ACP due to the presence of two conserved ACP-binding residues, Arg_129_ and Arg_172_ (Supplementary Figure 1), as these residues have been shown to interact with ACP in other FabG models^76^. Overall, analysis of the FabG sequence, structure, and enzymatic assays, suggests FabG (ABO11256.2) is indeed a functional, and is likely the primary 3-oxoacyl-ACP reductase of *A. baumannii*.

### Structural analysis of *Ab*SDR; a short chain dehydrogenase/reductase

**Figure 3:**
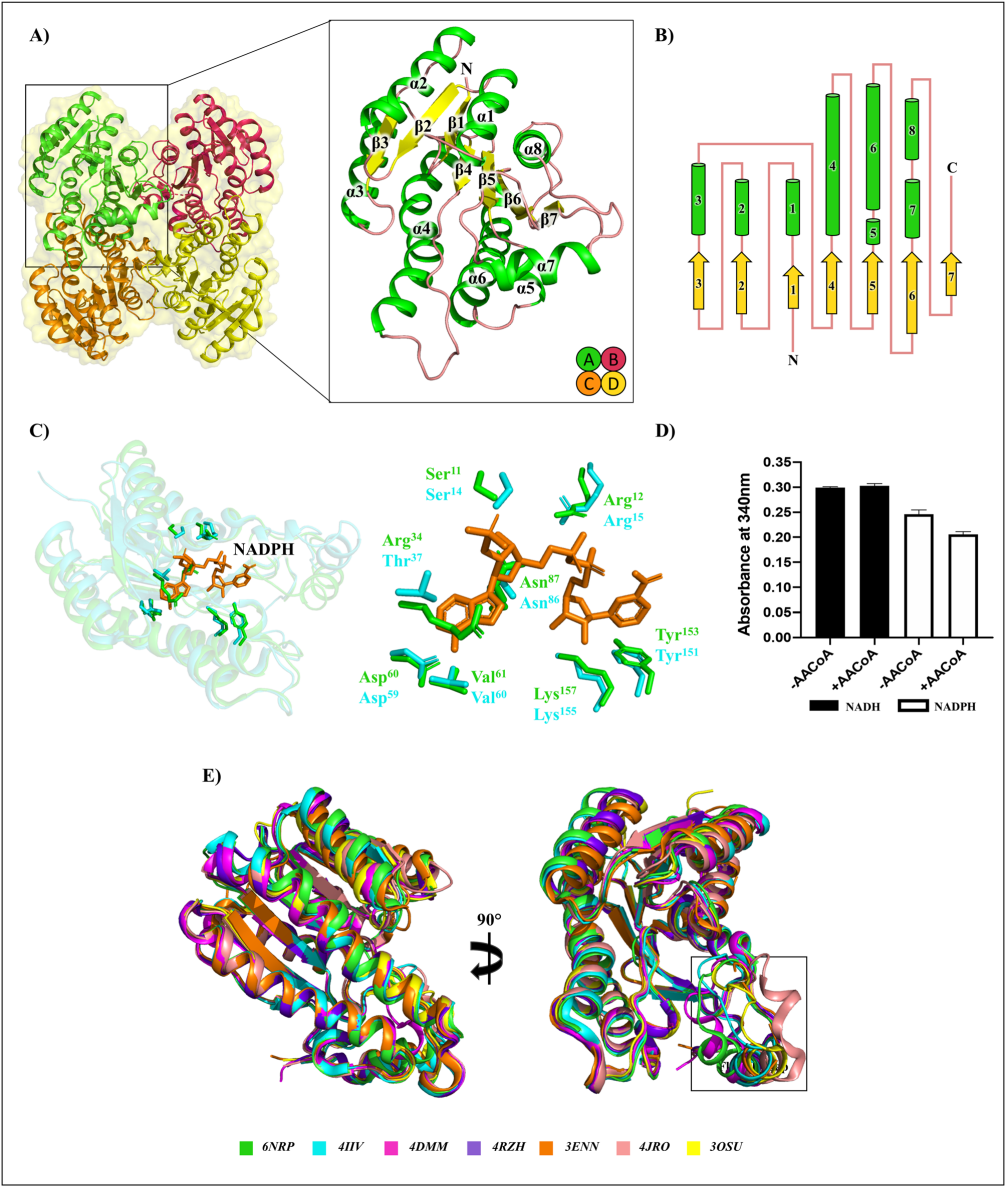
Structure of *Ab*SDR (ABO10977.2) (**A**) The quaternary assembly and protomer displaying the secondary structure. This model was deposited in the PDB under the code 6NRP. (**B**) Topology diagram of protomer assembly with loops (salmon), α-helices (green), and β-sheets (yellow). (**C**) Superimposition of FabG (cyan) and *Ab*SDR (green) with NADPH (orange) at the active site. (**D**) FabG enzyme activity assay using acetoacetyl-CoA substrate. (**E**) Superimposition of structurally similar SDR proteins determined through the DALI server. 6NRP short chain dehydrogenase/reductase from *A. baumannii* (green, this study), 4IIV 3-oxoacyl-ACP reductase *E. coli * strain CFT073 (cyan, *unpublished*), 4DMM 3-oxoacyl-ACP reductase from *Synechococcus elongatus* PCC 7942 in complex with NADP (magenta, *unpublished*), 4RZH 3-oxoacyl-ACP reductase from *Synechocystis* sp. PCC 6803 (purple, Liu et al.^43^), 3ENN glucose/ribitol dehydrogenase from *Brucella melitensis* (orange, *unpublished*), 4JRO 3-oxoacyl-ACP reductase *Listeria monocytogenes* in complex with NADP^+^ (salmon, *unpublished*), 3OSU 3-oxoacyl-ACP reductase from *Staphylococcus aureus* (yellow, *unpublished*). The black box indicates the SDR flexible capping region. All figure images were generated/drawn by the authors.

Another putative *A. baumannii* gene annotated as a putative 3-oxoacyl-ACP reductase was *Ab*SDR (ABO10977.2), however, unlike FabG, it was not conserved across all species examined. *Ab*SDR is a classical SDR enzyme, where our bioinformatics analysis revealed this protein to harbor a conserved domain belonging to the FabG_rel super family (cl36988, E-value; 3.56e-127) rather than the BKR_SDR_c subgroup of the NADB_Rossman super family that includes other FabG proteins, including *A. baumannii* FabG (cd05333, E-value; 8.57e-121). We therefore solved the crystal structure to determine if features of this protein were consistent with those for a typical 3-oxoacyl-ACP reductase. The best quality diffraction data for *Ab*SDR was collected on the Australian Synchrotron MX2 beamline to a resolution of 1.9 Å. The crystal was indexed in P 3_1_ 2 1 with cell lengths (Å) *a* = 89.48*, b* = 89.48*, c* = 239.47 and the phases solved using molecular replacement with PDB:4IIU (60% sequence identity; *unpublished*). The final model exhibited good stereochemistry and an overall R_work_ = 0.17 and R_free_ = 0.19 (Table 1). Each protomer displays a Rossmann fold (Fig. 3A), and a schematic view of structural topology (Fig. 3B) highlights a central β-sheet displaying a β3-β2-β1-β4-β5-β6-β7 pattern. This β-sheet is flanked by two groups of helices (α3, α4, α5, α6 and α2, α1, α8). Interestingly, *Ab*SDR contains only one alpha helix (α7) in the flexible capping subdomain, which is conserved in SDR proteins. Alternatively, our FabG protein displays two helices α7 and α8 in the same region. This subdomain is responsible for dimerization (generating a dimer of dimers [tetramer]) which is involved in the formation of a tunnel to the active site^51^.

Overall, four protomers were present in the crystallographic asymmetric unit, each displaying high structural homology (greatest r.m.s.d of 0.158 Å, Fig. 3A). The tetramer is very similar to *A. baumannii* FabG and the biological unit is confirmed by gel filtration elution volume (tetramer ≈ 100 kDa) and SDS-PAGE (protomer ≈ 26 kDa). To further confirm that the homotetramer observed in the asymmetric unit was indeed representative of the biological unit, we performed PDBsum analysis (Supplementary Table 4)^42^. The quaternary structure showed a homotetramer with two interfaces: the α8 and β7 of opposing antiparallel protomers at one interface (A/B interface area 1363.5 Å, 1 salt bridge, 12 hydrogen bonds, and 184 non-bonded contacts) and the α4 and α6 helices of opposing antiparallel protomers at the other (A/C interface area 1642.5 Å, 4 salt bridges, and 22 hydrogen bonds, and 212 non-bonded contacts). A weak interaction is observed between the diagonal B/C interface, with 5 non-bonded contacts.

In terms of catalytic ability, the active site residues are conserved, and closely resemble that of our FabG structure. At the active site, three SDR catalytic residues, Ser_140_-Tyr_153_-Lys_157_ are present, and Ser_111_ creates a kink in the α4 helix. Superimposition shows that similar to FabG, the active site architecture is in an ‘active’ conformation, even without the presence of coenzyme suggesting conformational change may not be necessary for coenzyme binding (Fig. 3C). By analogy, and due to the conserved nature of the active site residues, we assume a similar catalytic reaction to that already determined for SDR proteins^26^. *Ab*SDR has two basic residues Arg_12_ and Arg_34_ that suggests specificity towards a NADPH coenzyme rather than NADH, and classification in the cP3 coenzyme-binding subfamily^38,63^. We investigated the binding site to assess ability of *Ab*SDR to bind NADPH, by superimposing *Ab*SDR with our NADPH bound FabG model, revealing the two NADPH determining basic residues, Arg_12_ and Arg_34_, alongside other conserved residues involved in coenzyme binding (Fig. 3C). Functional assays did not show clear activity, unlike that for FabG where all available NADPH was converted to NADH in the reaction (Fig. 3D). In terms of ability to bind ACP, *Ab*SDR residues are conserved at the ACP-coordination site, including Lys_131_ and Arg_174_ (Supplementary Figure 1).

### Comparative analyses of *A. baumannii* FabG and *Ab*SDR

Since our enzyme activity analysis revealed limited NADPH conversion by *Ab*SDR, we searched for closely related structures. Comparison of our structure to others in the PDB was conducted using the DALI server^33^ and a protomer from each is superimposed in (Fig. 3E). The highest similarity (r.m.s.d for 241 residues) was observed for a putative 3-oxoacyl-ACP reductase from *Escherichia coli* strain CFT073 complexed with NADP^+^ (PDB: 4IIV, r.m.s.d = 1.0, *unpublished*), 3-oxoacyl-ACP reductase from *Synechococcus elongatus* PCC 7942 in complex with NADP (PDB: 4DMM, r.m.s.d = 1.3, *unpublished*), FabG from *Synechocystis sp.* PCC 6803 (PDB: 4RZH, r.m.s.d = 1.3, Liu et al.^43^), glucose/ribitol dehydrogenase from *Brucella melitensis* (PDB: 3ENN, r.m.s.d = 1.4, *unpublished*), 3-oxoacyl-ACP reductase *Listeria monocytogenes* in complex with NADP^+^ (PDB: 4JRO, r.m.s.d = 1.5, *unpublished*), and 3-oxoacyl-ACP reductase, FabG, from *Staphylococcus aureus* (PDB: 3OSU, r.m.s.d = 1.6, *unpublished*). While the most closely related structures are annotated as FabG enzymes, majority are unpublished and without supporting biochemical assays, and therefore, FASII functions remain putative.

Previous analyses of other FabG-like SDR enzymes have shown no activity in vitro in *A. baumannii*^16^ and roles in steroid metabolism in *M. tuberculosis*^71^. As SDR proteins maintain conserved structural features, whilst performing an array of functions, it can be difficult to assign biological function based on their structure or sequence. FabG2 from *Xanthomonas campestris* (a plant-associated bacterial pathogen) is unable to catalyze the reduction of short-chain (3-oxoacyl-ACP) substrates in an initial FabG reaction, however, can participate when longer substrate chains (≥ C_8_) are available^35^. FabG3 from *X. campestris,* whilst functional against acyl chains of various lengths, showed lowered reductive activity compared to functional FabG1 and was not essential for fatty acid synthesis, rather likely involved in xanthomonadin synthesis^73^. FabG1 and FabG3 of *X. campestris* contain the necessary hallmarks of a FabG-catalytic triad, N-terminal coenzyme binding motif, and an ability to bind ACP^35,73^. *X. campestris* belong to the same class of gamma-proteobacteria as *A. baumannii*, and as such there may be potential that *Ab*SDR performs a similar ancillary function within fatty acid biosynthesis. We suggest the annotation for *Ab*SDR remains as a putative 3-oxoacyl-ACP reductase.

### *A. baumannii* HMwFabG: A conserved high molecular weight 3-oxoacyl-ACP reductase

A much larger protein (463 aa) annotated as a 3-oxoacyl-ACP reductase was revealed during initial *A. baumannii* sequence analysis. High molecular weight 3-oxoacyl-ACP reductases are noted in Proteobacteria and Actinobacteria, however, only one crystal structure for a HMwFabG has been characterized to date: *Mt*FabG4 PDB: 3M1L^19^. We therefore investigated the structure of this protein, to better understand this class of enzymes and its potential role in *A. baumannii*. After successful expression and purification of the recombinant protein, crystallography trials yielded two crystal forms with different space groups. These structures were modelled using molecular replacement with starting model PDB: 5VP5 (48% sequence identity, *unpublished*). The first structure was resolved to a resolution of 1.8 Å, with P1 symmetry, and a R_work_ = 0.18 and R_free_ = 0.21 (designated as crystal form 1 or CF1). The structure from the second crystal form was resolved to a resolution of 1.65 Å, modelled in the P 1 2_1_ 1 space group, had overall R_work_ = 0.2 and R_free_ = 0.24, (and was designated crystal form 2 or CF2). All data collection and refinement statistics for both structures are listed in Table 1. The CF1 model showed poor electron density for residues 1–25 in chain A and 1–22 in chain B and for this reason, these regions could not be modelled. For CF2, N-terminal residues could be built in chain A and show a bent helix structure (residues 4–22), unlike *Mt*FabG4 where this region was truncated (1–16) to assist with protein solubility during expression and purification^19^. Chain B of CF2 had regions of flexibility and poor electron density between residues 1–22 and 405–413 where it could not be built. As the models were structurally very similar, (r.m.s.d between protomers of the two models was 0.179 Å), CF2 (PDB: 6UUT) was used for the remaining analysis.

**Figure 4:**
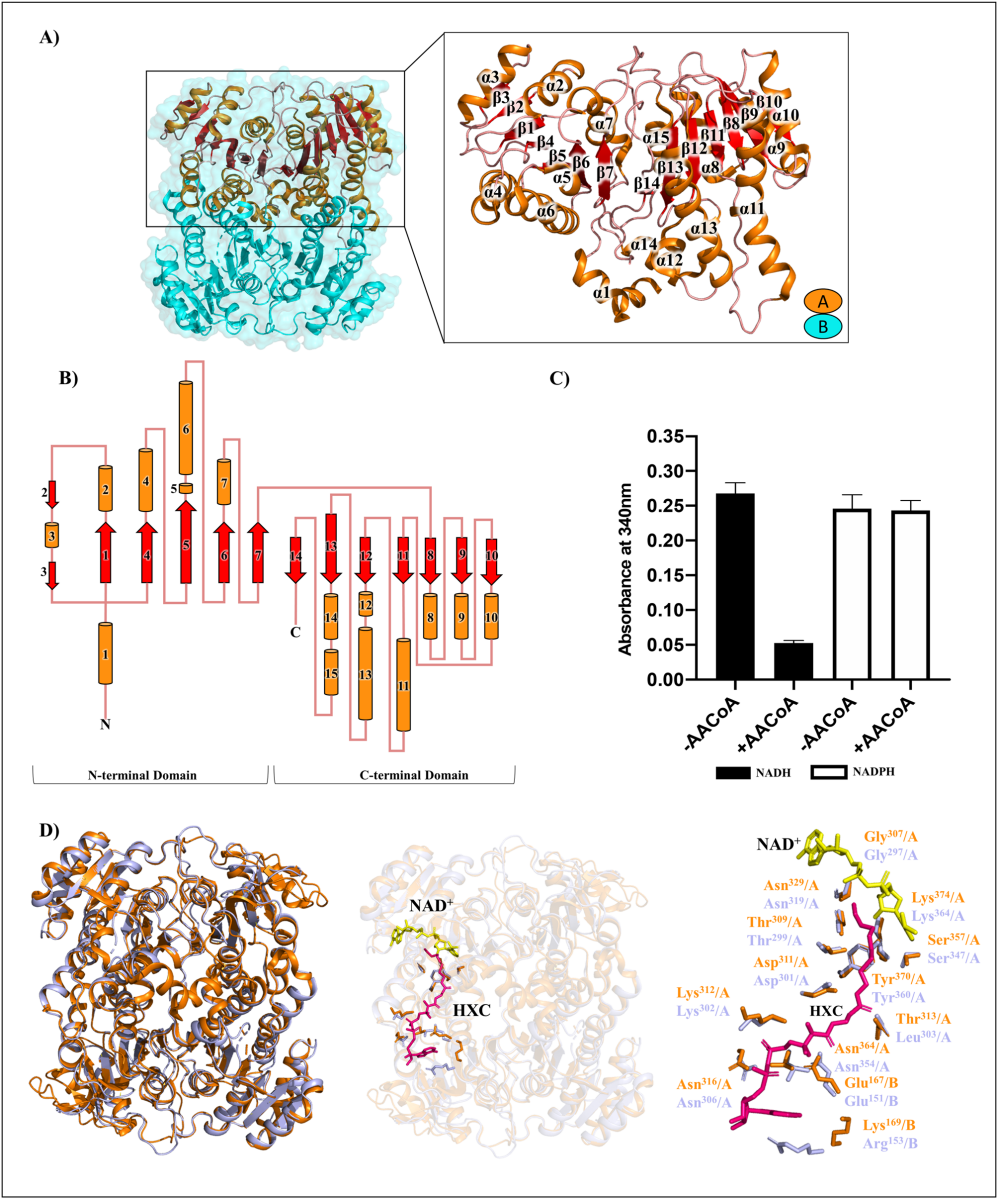
Structure of *A. baumannii* HMwFabG (ABO12488.2) (**A**) The dimeric quaternary assembly and protomer displaying the flavodoxin-like N-terminal fold and Rossmann C-terminal fold. Models were deposited in the PDB under the code 6UUV and 6UUT. (**B**) Topology diagram of protomer assembly with loops (salmon), α-helices (orange), and β-sheets (red). (**C**) Enzyme activity of HMwFabG with acetoacetyl-CoA. (**D**) Superimposition of *A. baumannii* HMwFabG (orange) and *M. tuberculosis* FabG4 (purple, PDB: 3V1U) (left panel), with hexanoyl-CoA (HXC, magenta) and NAD^+^ (yellow) (middle panel), and superimposed active site showing conserved sidechains, coenzyme, and substrate (right panel). All figure images were generated/drawn by the authors.

The crystallographic asymmetric unit contains two protomers (Fig. 4A). Each protomer has two domains; an N-terminal domain: β2-β3-β1-β4-β5-β6-β7 and C-terminal domain: β14-β13-β12-β11-β8-β9-β10 (Fig. 4B). The N-terminal domain displays a flavodoxin-like fold important for dimeric chain interaction and orientation as previously described^19^. The C-terminal domain resembles a typical FabG enzyme Rossmann fold with a β-α-β cross-over region, fingerprint coenzyme binding residues and catalytic residues. In terms of quaternary structure, both models complemented elution traces from gel filtration suggesting HMwFabG is a dimer in solution with protomers an approximate size of ≈ 48 kDa, also confirmed by SDS-PAGE. This was further confirmed through calculation of interface statistics using PDBsum (Supplementary Table 5)^42^. The average total interface area was 3057 Å^2^, and had 4 salt bridges, 32 hydrogen bonds, and 333 non-bonded contacts. These interactions create an antiparallel homodimer formed between helices at the N-terminal domain of one protomer with the C-terminal domain of the opposing protomer. That is, α4 and α6 of the N-terminal domain interact with α11 and α13 on the C-terminal domain opposing protomer. Superimposition of the two structures also reveals an extra β2-α3-β3 motif following the α2 helix of our structure, where a loop region (residues 73–80 not modelled) is seen in *Mt*FabG4^20^. Overall, the general structures of HMwFabG proteins are conserved, and the catalytic C-terminal domain closely resembles the machinery used by classical low molecular weight FabG enzymes. Within the C-terminal domain of HMwFabG is the Ser_357_-Tyr_370_-Lys_374_ catalytic triad and Asn_329_ creates a kink in the α10 helix important for proton replenishment and this domain closely resembles the structure of a classic 3-oxoacyl-ACP reductase, including our FabG.

Preference for NADH coenzyme is determined by Asp_256_ which is suggested to decrease available volume of the 2′phosphate accommodating region, thereby providing an unfavourable binding environment for the larger NADP(H) coenzyme^20^. Our enzyme assays showed clear preference for NADH as the catalytic coenzyme (Fig. 4C). Attempts to co-crystallize HMwFabG and NADH were unsuccessful. Since our structure was homologous with the *Mt*FabG4, which also prefers NADH, we compared the two models to analyse the ability of HMwFabG to accommodate coenzyme and substrate^20^ (Fig. 4D). Superimposition with *Mt*FabG4 (49% sequence identity) NAD^+^ and HXC ligands reveals our structure possesses an active site that can accommodate coenzyme and substrate, and highlights key residues involved with interactions between coenzyme and substrate (Fig. 4D)^20^. These residues are conserved in HMwFabG, with the exception of Thr_313_ and Lys_169_. Lys_169_, shows significant sidechain movement, however, conformational rearrangement at this site has been noted previously^20^.

Essential ACP binding residues are identified for low molecular weight FabG^76^ and HMwFabG^20^. In our HMwFabG, these residues translate to Arg_127_ and Lys_166_ which contribute to a positively charged cluster of residues at the entrance to the active site that enables ACP binding (Supplementary Figure 1). Both our structural and functional data suggest that HMwFabG has the capacity to participate as a reductase in the FASII pathway of *A. baumannii*.

### HMwFabG is required for pellicle formation, motility and growth under nutrient rich conditions

**Figure 5:**
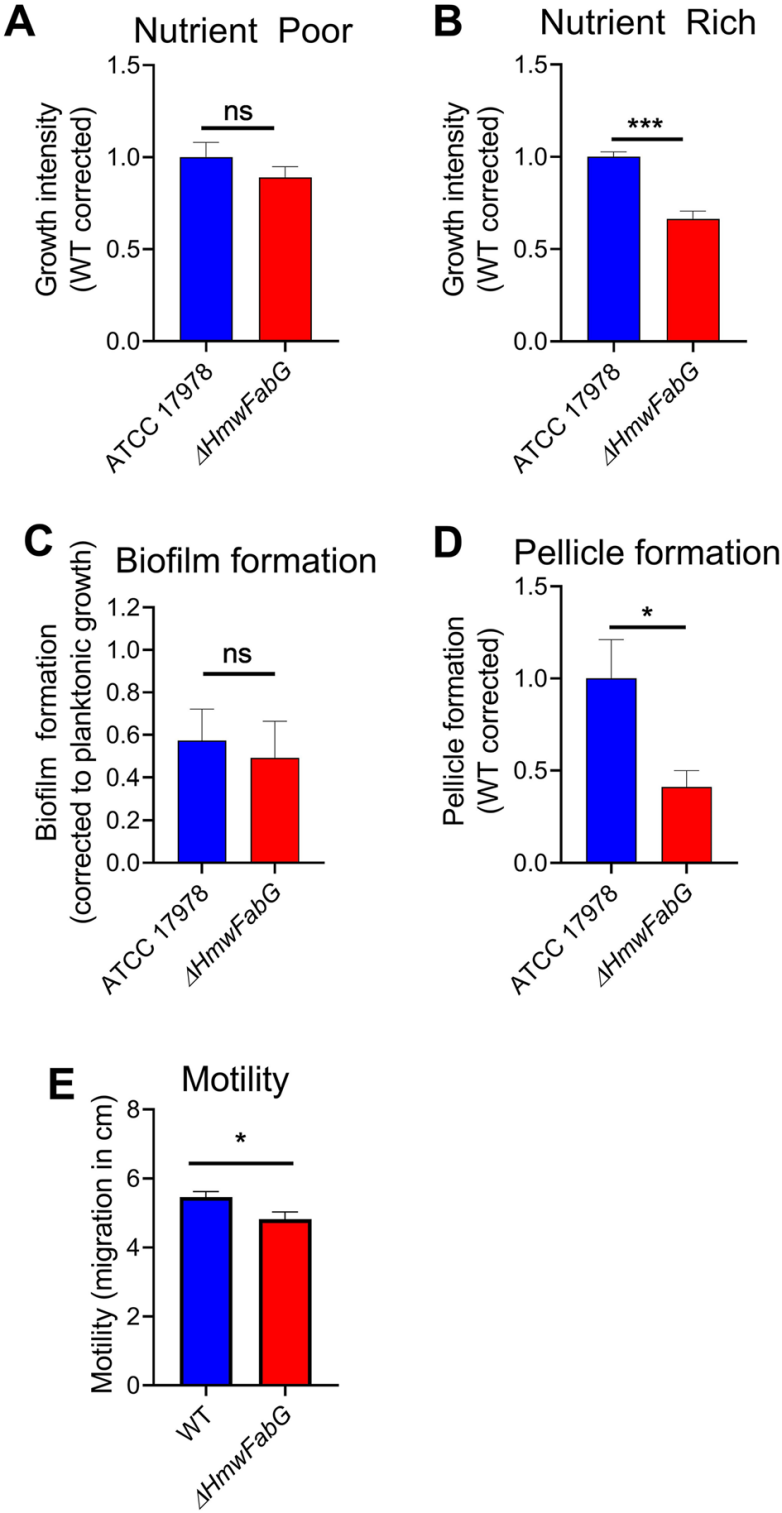
HMwFabG is important for pellicle formation, motility and growth under nutrient rich conditions. Growth of ATCC 17978 and *ΔAbHMwfabG* in nutrient poor conditions (**A**), and nutrient rich conditions (**B**). Biofilm (OD_590_/OD_600_) (**C**), pellicle (**D**) and motility (**E**) of ATCC 17978 and *ΔAbHMwfabG.* For all panels, results are the mean ± standard error of the mean (SEM) from at least biological quadruplicates. Statistical analyses were performed using an unpaired, two-tailed Student’s *t*-test; * *p* < 0.05, *** *p* < 0.001, ns = not significant.

Previously, *Mt*FabG4 was deemed essential for Mycobacterial growth in Roisin’s minimal media^10^. Given NADH is a lower energy molecule relative to that of NADPH and is largely associated with catabolic pathways, a role for HMwFabG enzymes under nutrient limited environments has been proposed^18^. To examine the role of *Ab*HMwFabG, the HMwFabG from the well-studied laboratory reference strain ATCC 17978 (A1S_2061) was deleted by allelic replacement, generating ∆*AbHMwfabG*. The ability of ∆*AbHMwfabG* to grow in M9 minimal media (nutrient poor) or LB media (nutrient rich) were assessed. Interestingly, unlike that of *Mt*FabG4, no significant differences were identified for ∆*AbHMwfabG* compared to WT in nutrient poor media (Fig. 5A), whilst *∆AbHMwfabG* was significantly impaired in its ability to grow in nutrient rich conditions (Fig. 5B).

The HMwFabG encoded in *P. aeruginosa* PAO1 (PA4786) has been shown to be involved in the production of a quorum sensing signalling molecule^30^, which can impact lifestyle changes such as switching to a sessile growth state. To examine the impact HMwFabG may have on *A. baumannii* lifestyles; motility, biofilm and pellicle formation were examined. No significant differences were observed in biofilm formation (Fig. 5C), whilst Δ*AbHMwfabG* was significantly impaired in its ability to form a pellicle (Fig. 5D). Pellicles are communities of bacteria that form at the air–liquid interface and provide a favourable environment for strict aerobes such as *A. baumannii* as they support efficient oxygen uptake from the air and nutrient acquisition from the media below^29,46^. Similar to biofilms at the solid–liquid interface, bacteria in pellicles are encased in an extracellular polymeric matrix comprised of various molecules including exopolysaccharides, proteins, extracellular DNA and lipids. The matrix is known to enhance motility, horizontal gene transfer and promote cell-to-cell signalling^44^. Accordingly, motility of the Δ*AbHMwfabG* mutants was significantly lower compared to that of the parental strain (Fig. 5E). A proteomic analysis revealed *Ab*HMwFabG to be up-regulated in 4-day pellicles compared to cells grown in the planktonic state^40^, which is in support of our findings for a role of *Ab*HMwFabG in pellicle biogenesis. *Ab*HMwFabG may aid in the synthesis of fatty acids that constitute the pellicle matrix or be involved in the production of quorum sensing molecules that *A. baumannii* produces, such as *N*-hydroxydodecanoyl-L- homoserine lactone, which facilitates efficient cell-to-cell communication in this sessile growth mode. To confirm that the *ΔAbHMwfabG* mutant was responsible for pellicle formation, we sequenced the complete genome of the *ΔAbHMwfabG* mutant and did not identify any mutations (in addition to the target gene) that are likely to impact *A. baumannii*. The only notable mutation we identified included a premature stop codon in a hypothetical gene (ACX60_RS15975). However, upon further examination of other *A. baumannii* genomes, this gene was found to readily accumulate premature stop codons (e.g. in *A. baumannii* strain AB0057). Hence, a contribution to the phenotypes reported is highly unlikely.

Overall, the biological advantage of *A. baumannii* possessing a secondary HMwFabG enzyme is currently unknown. In *Mycobacterium* it is hypothesized that this enzyme is required for survival in unfavourable conditions^18^. Upregulation of *Mt*FabG4 was observed when cells were treated with the aminoglycoside, streptomycin^57^. Interestingly, RNA sequencing of colistin treated *A. baumannii* revealed expression of *HMwFabG* was upregulated by 5.28- and 4.0-fold (log_2_) after 15 min and 1 h shock treatments, respectively^32^. Furthermore, transcription of the low molecular weight *fabG* was downregulated 3.73- and 3.03-fold after 15 min and 1 h colistin shock treatments, respectively^32^. These findings suggest that *A. baumannii* has the capacity to switch between FabG homologs, which is dependent on environmental factors. However, drug resistance analyses of ATCC 17978 and the *∆AbHMwfabG* mutant by defining the minimal inhibitory concentration to colistin, chloramphenicol, gentamicin or streptomycin did not reveal changes greater than two-fold (Supplementary Table 6).

Recent advances in the development of inhibitors against *Mt*FabG4 are reviewed comprehensively by Dutta^18^. Successful inhibitors targeting *Mt*FabG4 NADH binding sites include triazole linked polyphenol or polyphenol-aminobenzene hybrids^7,8^. Dual inhibitors of *Mt*FabG4 and HtdX, another enzyme that belongs to the same operon in *M. tuberculosis* and is presumed to play a role in fatty acid metabolism, bind at catalytic loops^7,8^. A specifically designed synthetic inhibitor of *Mt*FabG4, S-S006-830, found two putative binding regions; the first near the substrate/coenzyme binding sites in the C-terminal domain and the other at a smaller pocket on the N-terminal domain^58^. Given the structural similarity between MtFabG4 and HMwFabG of *A. baumannii*, these inhibitors may also provide a new opportunity to treat drug-resistant *A. baumannii* and should be examined. The type of inhibitors designed would likely determine whether these act as narrow or broad-spectrum therapeutics. Inhibitors that act within conserved binding site regions of FabG would likely inhibit all FabG isoforms, however, may also affect other bacterium including normal flora, an off-target effect similar to most antibiotics in current use. Inhibitors that specifically target one FabG isoform (for example a HMwFabG N-terminal domain-targeting drug) would likely be narrow spectrum but may allow *A. baumannii* to switch preference to another FabG. In this case, these inhibitors may be useful under specific environmental conditions, though the conditions for lifestyle dependent FabG switching remains largely unknown. Moreover, the use of dual cocktail inhibitors targeting individual FabG isoforms may also be a potential therapeutic option.

## Conclusion

In this study, we investigated the FASII pathway of *A. baumannii*, providing insights into potential 3-oxoacyl-ACP reductase (FabG) homologs. Our bioinformatics analysis identified potential SDR candidates that harboured necessary catalytic residues and binding motifs and thus may function as FabG. We provide high quality crystal structures for three enzymes: a functional low molecular weight FabG, a putative FabG protein, *Ab*SDR, and a HMwFabG. We have provided evidence that the HMwFabG of *A. baumannii* is required for optimal growth in nutrient rich conditions, motility and pellicle biogenesis. The exact function and pathways involved in HMwFabG activity remains elusive but may contribute to its survival and success as a pathogen. *A. baumannii* is a pathogen of extreme concern in healthcare institutions where nosocomial infections are increasingly difficult to treat due to antimicrobial resistance. Specific targeting of the FabG or HMwFabG enzymes in *Acinetobacter* may be a promising new focus for antimicrobial design.

## Supplementary Information

Supplementary Information

## References

[1] Adams FG, Stroeher UH, Hassan KA, Marri S, Brown MH (2018). Resistance to pentamidine is mediated by AdeAB, regulated by AdeRS, and influenced by growth conditions in *Acinetobacter baumannii* ATCC 17978. PLoS ONE.

[2] Adams PD, Afonine PV, Bunkóczi G, Chen VB, Davis IW, Echols N, Zwart PH (2010). PHENIX: a comprehensive python-based system for macromolecular structure solution. Acta Crystallogr. D Biol. Crystallogr..

[3] Alquethamy SF, Adams FG, Naidu V, Khorvash M, Pederick VG, Zang M, Eijkelkamp BA (2020). The role of zinc efflux during *Acinetobacter baumannii* Infection. ACS Infect. Dis..

[4] Altschul SF, Madden TL, Schäffer AA, Zhang J, Zhang Z, Miller W, Lipman DJ (1997). Gapped BLAST and PSI-BLAST: a new generation of protein database search programs. Nucleic Acids Res..

[5] Aragão D, Aishima J, Cherukuvada H, Clarken R, Clift M, Cowieson NP, Caradoc-Davies TT (2018). MX2: a high-flux undulator microfocus beamline serving both the chemical and macromolecular crystallography communities at the Australian Synchrotron. J. Synchrotron Radiat..

[6] Asturias FJ, Chadick JZ, Cheung IK, Stark H, Witkowski A, Joshi AK, Smith S (2005). Structure and molecular organization of mammalian fatty acid synthase. Nat. Struct. Mol. Biol..

[7] Banerjee DR, Biswas R, Das AK, Basak A (2015). Design, synthesis and characterization of dual inhibitors against new targets FabG4 and HtdX of *Mycobacterium tuberculosis*. Eur. J. Med. Chem..

[8] Banerjee DR, Senapati K, Biswas R, Das AK, Basak A (2015). Inhibition of *M. tuberculosis* β-ketoacyl CoA reductase FabG4 (Rv0242c) by triazole linked polyphenol–aminobenzene hybrids: comparison with the corresponding gallate counterparts. Bioorgan. Med. Chem. Lett..

[9] Battye TGG, Kontogiannis L, Johnson O, Powell HR, Leslie AGW (2011). iMOSFLM: a new graphical interface for diffraction-image processing with MOSFLM. Acta Crystallogr. D Biol. Crystallogr..

[10] Beste DJ, Espasa M, Bonde B, Kierzek AM, Stewart GR, McFadden J (2009). The genetic requirements for fast and slow growth in mycobacteria. PLoS ONE.

[11] Campbell JW, Cronan JE (2001). Bacterial fatty acid biosynthesis: targets for antibacterial drug discovery. Annu. Rev. Microbiol..

[12] CDC. (2019). Antibiotic Resistance Threats in the United States, 2019. Retrieved from https://www.cdc.gov/drugresistance/biggest-threats.html#acine

[13] Cohen-Gonsaud M, Ducasse S, Hoh F, Zerbib D, Labesse G, Quemard AK (2002). Crystal structure of MabA from *Mycobacterium tuberculosis*, a reductase involved in long-chain fatty acid biosynthesis. J. Mol. Biol..

[14] Cole ST, Brosch R, Parkhill J, Garnier T, Churcher C, Harris D, Barrell BG (1998). Deciphering the biology of *Mycobacterium tuberculosis* from the complete genome sequence. Nature.

[15] Cowieson NP, Aragao D, Clift M, Ericsson DJ, Gee C, Harrop SJ, Caradoc-Davies T (2015). MX1: a bending-magnet crystallography beamline serving both chemical and macromolecular crystallography communities at the Australian Synchrotron. J. Synchrotron Radiat..

[16] Cross EM, Aragão D, Smith KM, Shaw KI, Nanson JD, Raidal SR, Forwood JK (2019). Structural characterization of a short-chain dehydrogenase/reductase from multi-drug resistant *Acinetobacter baumannii*. Biochem. Biophys. Res. Commun..

[17] Darling ACE, Mau B, Blattner FR, Perna NT (2004). Mauve: multiple alignment of conserved genomic sequence with rearrangements. Genome Res..

[18] Dutta D (2018). Advance in research on *Mycobacterium tuberculosis* FabG4 and its inhibitor. Front. Microbiol..

[19] Dutta D, Bhattacharyya S, Mukherjee S, Saha B, Das AK (2011). Crystal structure of FabG4 from *Mycobacterium tuberculosis* reveals the importance of C-terminal residues in ketoreductase activity. J. Struct. Biol..

[20] Dutta D, Bhattacharyya S, Roychowdhury A, Biswas R, Das AK (2013). Crystal structure of hexanoyl-CoA bound to beta-ketoacyl reductase FabG4 of *Mycobacterium tuberculosis*. Biochem. J..

[21] Eijkelkamp BA, Stroeher UH, Hassan KA, Papadimitrious MS, Paulsen IT, Brown MH, Lo R (2011). Adherence and motility characteristics of clinical *Acinetobacter baumannii* isolates. FEMS Microbiol. Lett..

[22] Emsley P, Cowtan K (2004). Coot: model-building tools for molecular graphics. Acta Crystallogr. Sect. D.

[23] Evans PR, Murshudov GN (2013). How good are my data and what is the resolution?. Acta Crystallogr. D Biol. Crystallogr..

[24] Farrugia DN, Elbourne LDH, Hassan KA, Eijkelkamp BA, Tetu SG, Brown MH, Paulsen IT (2013). The complete genome and phenome of a community-acquired *Acinetobacter baumannii*. PLoS ONE.

[25] Feng S-X, Ma J-C, Yang J, Hu Z, Zhu L, Bi H-K, Wang H-H (2015). Ralstonia solanacearum fatty acid composition is determined by interaction of two 3-ketoacyl-acyl carrier protein reductases encoded on separate replicons. BMC Microbiol..

[26] Filling C, Berndt KD, Benach J, Knapp S, Prozorovski T, Nordling E, Oppermann U (2002). Critical residues for structure and catalysis in short-chain dehydrogenases/reductases. J. Biol. Chem..

[27] Fournier P-E, Vallenet D, Barbe V, Audic S, Ogata H, Poirel L, Claverie J-M (2006). Comparative genomics of multidrug resistance in *Acinetobacter baumannii*. PLoS Genet..

[28] Gallagher LA, Ramage E, Weiss EJ, Radey M, Hayden HS, Held KG, Manoil C (2015). Resources for genetic and genomic analysis of emerging pathogen *Acinetobacter baumannii*. J. Bacteriol..

[29] Giles SK, Stroeher UH, Eijkelkamp BA, Brown MH (2015). Identification of genes essential for pellicle formation in *Acinetobacter baumannii*. BMC Microbiol..

[30] Guo QQ, Zhang WB, Zhang C, Song YL, Liao YL, Ma JC, Wang HH (2019). Characterization of 3-oxacyl-acyl carrier protein reductase homolog genes in pseudomonas aeruginosa PAO1. Front. Microbiol..

[31] Harding CM, Hennon SW, Feldman MF (2017). Uncovering the mechanisms of *Acinetobacter baumannii* virulence. Nat. Rev. Microbiol..

[32] Henry R, Crane B, Powell D, Deveson Lucas D, Li Z, Aranda J, Li J (2015). The transcriptomic response of *Acinetobacter baumannii* to colistin and doripenem alone and in combination in an in vitro pharmacokinetics/pharmacodynamics model. J. Antimicrob. Chemother..

[33] Holm L, Laakso LM (2016). Dali server update. Nucleic Acids Res..

[34] Hou J, Zheng H, Chruszcz M, Zimmerman MD, Shumilin IA, Osinski T, Minor W (2016). Dissecting the structural elements for the activation of β-ketoacyl-(acyl carrier protein) reductase from *Vibrio cholerae*. J. Bacteriol..

[35] Hu Z, Dong H, Ma JC, Yu Y, Li KH, Guo QQ, Wang H (2018). Novel *Xanthomonas campestris* long-chain-specific 3-oxoacyl-acyl carrier protein reductase involved in diffusible signal factor synthesis. MBio.

[36] Huerta-Cepas J, Serra F, Bork P (2016). ETE 3: reconstruction, analysis, and visualization of phylogenomic data. Mol. Biol. Evol..

[37] Jörnvall H, Persson B, Krook M, Atrian S, Gonzalez-Duarte R, Jeffery J, Ghosh D (1995). Short-chain dehydrogenases/reductases (SDR). Biochemistry.

[38] Kallberg Y, Oppermann U, Jörnvall H, Persson B (2002). Short-chain dehydrogenases/reductases (SDRs). Eur. J. Biochem..

[39] Kanehisa M, Goto S (2000). KEGG: kyoto encyclopedia of genes and genomes. Nucleic Acids Res..

[40] Kentache T, Ben Abdelkrim A, Jouenne T, De E, Hardouin J (2017). Global dynamic proteome study of a pellicle-forming *Acinetobacter baumannii* strain. Mol. Cell. Proteomics.

[41] Kwon J, Mistry T, Ren J, Johnson ME, Mehboob S (2018). A novel series of enoyl reductase inhibitors targeting the ESKAPE pathogens, Staphylococcus aureus and *Acinetobacter baumannii*. Bioorg. Med. Chem..

[42] Laskowski RA, Jabłońska J, Pravda L, Vařeková RS, Thornton JM (2018). PDBsum: Structural summaries of PDB entries. Protein Sci. A Public. Protein Soc..

[43] Liu Y, Feng Y, Cao X, Li X, Xue S (2015). Structure-directed construction of a high-performance version of the enzyme FabG from the photosynthetic microorganismSynechocystis sp. PCC 6803. FEBS Lett..

[44] López D, Vlamakis H, Kolter R (2010). Biofilms. Cold Spring Harb. Perspect. Biol..

[45] Mao YH, Li F, Ma JC, Hu Z, Wang HH (2016). Sinorhizobium meliloti functionally replaces 3-oxoacyl-acyl carrier protein reductase (FabG) by overexpressing NodG during fatty acid synthesis. Mol. Plant Microbe Interact..

[46] Martí S, Rodríguez-Baño J, Catel-Ferreira M, Jouenne T, Vila J, Seifert H, Dé E (2011). Biofilm formation at the solid-liquid and air-liquid interfaces by Acinetobacter species. BMC. Res. Notes.

[47] McCoy AJ, Grosse-Kunstleve RW, Adams PD, Winn MD, Storoni LC, Read RJ (2007). Phaser crystallographic software. J. Appl. Crystallogr..

[48] Nanson JD, Forwood JK (2015). Structural characterisation of FabG from Yersinia pestis, a key component of bacterial fatty acid synthesis. PLoS ONE.

[49] O’Neill, J. (2016). *Review on Antimicrobial Resistance. Tackling drug resistant infections globally: final report and recommendations*. Retrieved from London, United Kingdom: https://amr-review.org/sites/default/files/160525_Final%20paper_with%20cover.pdf

[50] Peleg AY, Seifert H, Paterson DL (2008). *Acinetobacter baumannii*: emergence of a successful pathogen. Clin. Microbiol. Rev..

[51] Price AC, Zhang Y-M, Rock CO, White SW (2001). Structure of β-ketoacyl-[acyl carrier protein] reductase from *Escherichia coli*: negative cooperativity and its structural basis. Biochemistry.

[52] Price AC, Zhang Y-M, Rock CO, White SW (2004). Cofactor-induced conformational rearrangements establish a catalytically competent active site and a proton relay conduit in FabG. Structure.

[53] Rao NK, Nataraj V, Ravi M, Panchariya L, Palai K, Talapati SR, Antony T (2020). Ternary complex formation of AFN-1252 with *Acinetobacter baumannii* FabI and NADH: crystallographic and biochemical studies. Chem. Biol. Drug Des..

[54] Seol G, Park H, Ahn Y-J, Kang L-W (2019). Crystal structure of enoyl-acyl carrier protein reductase (FabI) from *Acinetobacter baumannii* as a target for broad-spectrum antibacterial drug. Bull. Korean Chem. Soc..

[55] Shah BS, Ashwood HE, Harrop SJ, Farrugia DN, Paulsen IT, Mabbutt BC (2018). Crystal structure of a UDP-GlcNAc epimerase for surface polysaccharide biosynthesis in *Acinetobacter baumannii*. PLoS ONE.

[56] Shah BS, Tetu SG, Harrop SJ, Paulsen IT, Mabbutt BC (2014). Structure of a short-chain dehydrogenase/reductase (SDR) within a genomic island from a clinical strain of *Acinetobacter baumannii*. Acta Crystallogr. Sect. F Struct. Biol. Commun..

[57] Sharma P, Kumar B, Singhal N, Katoch V, Venkatesan K, Chauhan D, Bisht D (2010). Streptomycin induced protein expression analysis in *Mycobacterium tuberculosis* by two-dimensional gel electrophoresis & mass spectrometry. Indian J. Med. Res..

[58] Singh P, Kumar SK, Maurya VK, Mehta BK, Ahmad H, Dwivedi AK, Sinha S (2017). S-enantiomer of the antitubercular compound S006–830 complements activity of frontline TB drugs and targets biogenesis of *Mycobacterium tuberculosis* cell envelope. ACS Omega.

[59] Sohn M-J, Zheng C-J, Kim W-G (2008). Macrolactin S, a new antibacterial agent with fab G-inhibitory activity from bacillus sp. AT28. J. Antibiot..

[60] Stamatakis A (2014). RAxML version 8: a tool for phylogenetic analysis and post-analysis of large phylogenies. Bioinformatics.

[61] Studier FW (2005). Protein production by auto-induction in high-density shaking cultures. Protein Expr. Purif..

[62] Tacconelli E, Carrara E, Savoldi A, Harbarth S, Mendelson M, Monnet DL, Zorzet A (2018). Discovery, research, and development of new antibiotics: the WHO priority list of antibiotic-resistant bacteria and tuberculosis. Lancet Infect. Dis..

[63] Tanaka N, Nonaka T, Nakanishi M, Deyashiki Y, Hara A, Mitsui Y (1996). Crystal structure of the ternary complex of mouse lung carbonyl reductase at 1.8 å resolution: the structural origin of coenzyme specificity in the short-chain dehydrogenase/reductase family. Structure.

[64] Tucker AT, Nowicki EM, Boll JM, Knauf GA, Burdis NC, Trent MS, Davies BW (2014). Defining gene-phenotype relationships in *Acinetobacter baumannii* through one-step chromosomal gene inactivation. MBio.

[65] Wang N, Ozer EA, Mandel MJ, Hauser AR (2014). Genome-Wide Identification of *Acinetobacter baumannii* genes necessary for persistence in the lung. MBio.

[66] Wang Y, Ma S (2013). Recent advances in inhibitors of bacterial fatty acid synthesis type II (FASII) system enzymes as potential antibacterial agents. ChemMedChem.

[67] Wickramasinghe SR, Inglis KA, Urch JE, Müller S, van Aalten DMF, Fairlamb AH (2006). Kinetic, inhibition and structural studies on 3-oxoacyl-ACP reductase from plasmodium falciparum, a key enzyme in fatty acid biosynthesis. Biochem. J..

[68] Wiegand I, Hilpert K, Hancock REW (2008). Agar and broth dilution methods to determine the minimal inhibitory concentration (MIC) of antimicrobial substances. Nat. Protoc..

[69] Wong D, Nielsen TB, Bonomo RA, Pantapalangkoor P, Luna B, Spellberg B (2017). Clinical and pathophysiological overview of Acinetobacter infections: a century of challenges. Clin. Microbiol. Rev..

[70] Xie R, Zhang XD, Zhao Q, Peng B, Zheng J (2018). Analysis of global prevalence of antibiotic resistance in *Acinetobacter baumannii* infections disclosed a faster increase in OECD countries. Emerg. Microbes Infect..

[71] Yang JK, Park MS, Waldo GS, Suh SW (2003). Directed evolution approach to a structural genomics project: Rv2002 from *Mycobacterium tuberculosis*. Proc. Natl. Acad. Sci. U.S.A..

[72] Yao J, Rock CO (2017). Bacterial fatty acid metabolism in modern antibiotic discovery. Biochim. Biophys. Acta (BBA) Mol. Cell Biol. of Lipids.

[73] Yu Y, Ma J, Guo Q, Ma J, Wang H (2019). A novel 3-oxoacyl-ACP reductase (FabG3) is involved in the *Xanthomonadin biosynthesis* of *Xanthomonas campestris* pv campestris. Mol. Plant Pathol..

[74] Zaccai NR, Carter LG, Berrow NS, Sainsbury S, Nettleship JE, Walter TS, Esnouf RM (2008). Crystal structure of a 3-oxoacyl-(acylcarrier protein) reductase (BA3989) from Bacillus anthracis at 2.4-Å resolution. Proteins Struct. Funct. Bioinform..

[75] Zhang Y-M, Rock CO (2004). Evaluation of epigallocatechin gallate and related plant polyphenols as inhibitors of the FabG and FabI reductases of bacterial type II fatty-acid synthase. J. Biol. Chem..

[76] Zhang Y-M, Wu B, Zheng J, Rock CO (2003). Key residues responsible for acyl carrier protein and β-ketoacyl-acyl carrier protein reductase (FabG) interaction. J. Biol. Chem..

