# Supplementary material for: Insights into Acinetobacter baumannii fatty acid synthesis 3-oxoacyl-ACP reductases
Source: Sci Rep. 2021 Mar 29;11:7050. doi: 10.1038/s41598-021-86400-1 (PMC8007833; doi:10.1038/s41598-021-86400-1)
Supplement: Supplementary file 1 — Supplementary Information [file 41598_2021_86400_MOESM1_ESM.docx]

# Insights into *Acinetobacter baumannii* fatty acid synthesis 3-oxacyl-ACP reductases

Emily M. Cross^a^, Felise G. Adams^b^, Jack K. Waters^b^, David Aragão^c,d^, Bart A. Eijkelkamp^b^, Jade K. Forwood^a^

**Supplementary Figure 1: Identification of *A. baumannii* SDRs with conserved ACP docking residues.** A multiple sequence alignment of representative *A. baumannii* SDRs that encode the typical catalytic motif ([ST]x_12_Yx_3_K) against FabG sequences with known 3-oxoacyl ACP reductive capacity (*E. coli* [K-12 FabG; NP_415611.1] and *P. aeruginosa* [PAO1 FabG; NP_251657.1]). Black shading represents positively charged residues at the conserved Arg-129 and Arg-172 position which facilitate ACP interaction (*E. coli* K-12 numbering). Conserved catalytic residues are coloured in blue, asterisk and colon symbols represent residues that are fully conserved and have similar physiochemical properties, respectively. Pink shading represents sequences that encode hydrophobic residues at the correct position. The numbers indicate the position of the last amino acids in the displayed sequences.


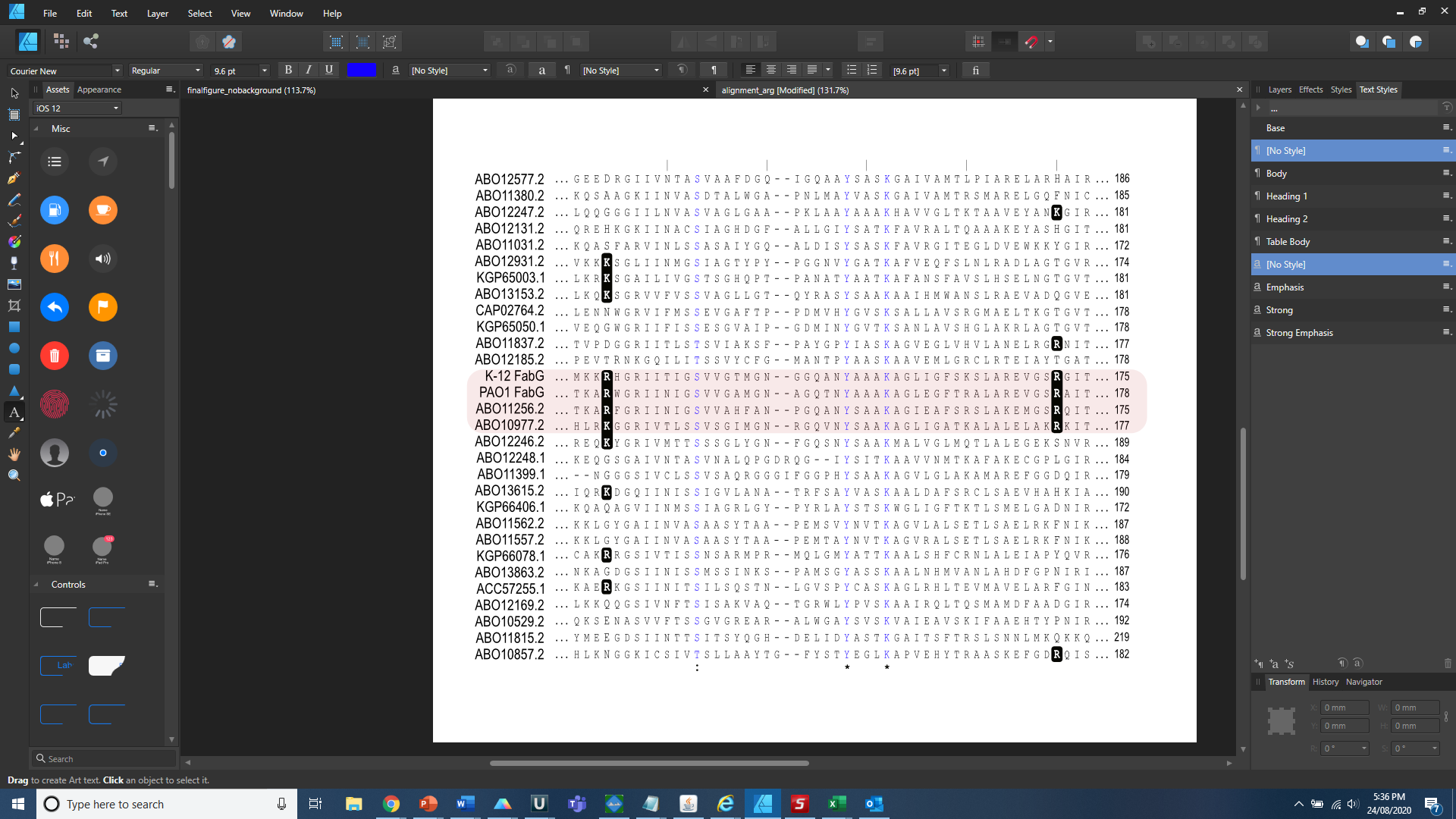


**Supplementary Table 1. Strains used in the study**

| **Name** | **Genotype or description** | **Reference / source** |
| --- | --- | --- |
| *A. baumannii* ATCC 17978 | Non international type clone; meninges isolate | ATCC |
| ∆*AbHMwfabG* | ATCC 17978 with kanamycin cassette insertion disruption in A1S_2061 | This study |

**Supplementary Table 2. Oligonucleotides used in the study**

| **Name** | **Sequence (5' - 3')** |
| --- | --- |
| A1S_2061 UFR F | CATTCTTCAGAAGCAAGC |
| A1S_2061 UFR R | GCGATTGTGTAGGCTGGAGCTGGACCCAAATGGAAGCGGAC |
| A1S_2061 DFR F | GTCCATATGAATATCCTCCTTCAGGCGAAGGTAAACCCAG |
| A1S_2061 DFR R | GCCCTCTCCTGCAAGAAATC |
| Kan F | GCGATTGTGTAGGCTGGAGCTG |
| Kan R | GTCCATATGAATATCCTCCTT |
| A1S_2061 NOL F | CCACAGAAGCACCATCAGTC |
| A1S_2061 NOL R | GGCTTAGTCGCTGCGACCACA |

**Supplementary Table 3.** Tetramer interface interactions for FabG

| **PDB 6UDS: A/B interface interactions** | | | | | | | |
| --- | --- | --- | --- | --- | --- | --- | --- |
| **Hydrogen Bonds** | | | | **Salt bridges** | | | |
| **Chain A** |  | **Chain B** | **Distance (Å)** | **Chain A** |  | **Chain B** | **Distance (Å)** |
| GLN173 [OE1] | <--> | ARG208 [NE] | 3.13 |  |  |  |  |
| GLN173 [NE2] | <--> | LEU206 [O] | 3.27 |  |  |  |  |
| PHE183 [O] | <--> | TYR229 [OH] | 3.22 |  |  |  |  |
| LEU206 [O] | <--> | GLN173 [NE2] | 2.94 |  |  |  |  |
| ARG208 [O] | <--> | TYR229 [OH] | 2.72 |  |  |  |  |
| ARG208 [NE] | <--> | GLN173 [OE1] | 2.94 |  |  |  |  |
| ARG208 [NH2] | <--> | GLY228 [O] | 3.01 |  |  |  |  |
| GLY228 [O] | <--> | ARG208 [NH2] | 3.11 |  |  |  |  |
| TYR229 [O] | <--> | GLY239 [N] | 2.85 |  |  |  |  |
| TYR229 [OH] | <--> | ARG208 [O] | 2.69 |  |  |  |  |
| GLY239 [N] | <--> | TYR229 [O] | 2.83 |  |  |  |  |
| ALA244[O] | <--> | VAL234 [N] | 3.32 |  |  |  |  |
|  |  |  |  |  |  |  |  |
| **A/C interface interactions** | | | | | | | |
| **Hydrogen Bonds** | | |  | **Salt Bridges** | | | |
| **Chain A** |  | **Chain C** | **Distance (Å)** | **Chain A** |  | **Chain C** | **Distance (Å)** |
| LEU95 [N] | <--> | GLU168 [OE1] | 2.91 | GLU100 [OE1] | <--> | LYS112 [NZ] | 2.75 |
| LEU95 [O] | <--> | LYS119 [NZ] | 2.74 | GLU100 [OE1] | <--> | ARG116 [NH2] | 2.83 |
| LEU96 [O] | <--> | LYS119 [NZ] | 2.84 | ASP104 [OD1] | <--> | LYS112 [NZ] | 2.77 |
| MET98 [O] | <--> | TYR115 [OH] | 2.84 | LYS112 [NZ] | <--> | GLU100 [OE1] | 2.92 |
| MET98 [O] | <--> | LYS119 [NZ] | 3.29 | LYS112 [NZ] | <--> | ASP104 [OD1] | 2.68 |
| GLU100 [OE1] | <--> | LYS112 [NZ] | 2.75 | ARG116 [NE] | <--> | GLU100 [OE2] | 2.87 |
| GLU100 [OE1] | <--> | ARG116 [NH2] | 2.83 |  |  |  |  |
| GLU100 [OE2] | <--> | ARG116 [NE] | 2.84 |  |  |  |  |
| ASP104 [OD1] | <--> | LYS112 [NZ] | 2.77 |  |  |  |  |
| LYS112 [NZ] | <--> | GLU100 [OE1] | 2.92 |  |  |  |  |
| LYS112 [NZ] | <--> | ASP104 [OD1] | 2.68 |  |  |  |  |
| TYR115 [OH] | <--> | MET98 [O] | 2.69 |  |  |  |  |
| ARG116 [NE] | <--> | GLU100 [OE2] | 2.87 |  |  |  |  |
| ARG116 [NH2] | <--> | GLU100 [OE1] | 2.90 |  |  |  |  |
| LYS119 [NZ] | <--> | LEU95 [O] | 2.92 |  |  |  |  |
| LYS119 [NZ] | <--> | LEU96 [O] | 2.73 |  |  |  |  |
| ALA141 [O] | <--> | ARG163 [NH2] | 2.98 |  |  |  |  |
| ALA144 [O] | <--> | LYS167 [NZ] | 2.48 |  |  |  |  |
| GLY147 [N] | <--> | GLU168 [OE2] | 2.65 |  |  |  |  |
| ARG163 [NH2] | <--> | ALA141 [O] | 3.20 |  |  |  |  |
| GLU168 [OE1] | <--> | LEU95 [N] | 2.79 |  |  |  |  |
| GLU168 [OE2] | <--> | GLY147 [N] | 2.72 |  |  |  |  |

| **PDB: 6NRP A/B interface interactions** | | | | | | | |
| --- | --- | --- | --- | --- | --- | --- | --- |
| **Hydrogen Bonds** | | | | **Salt bridges** | | | |
| **Chain A** |  | **Chain B** | **Distance (Å)** | **Chain A** |  | **Chain B** | **Distance (Å)** |
| LYS173 [NZ] | <--> | GLN206 [OE1] | 2.75 | GLU225 [OE2] | <--> | Lys219 [NZ] | 3.37 |
| ARG207 [O] | <--> | TYR228 [OH] | 2.70 |  |  |  |  |
| GLU213 [OE2] | <--> | SER227 [OG] | 2.65 |  |  |  |  |
| GLU225 [OE1] | <--> | SER216 [OG] | 2.84 |  |  |  |  |
| SER227 [OG] | <--> | GLU213 [OE2] | 2.60 |  |  |  |  |
| TYR228 [O] | <--> | GLY238 [N] | 2.93 |  |  |  |  |
| TYR228 [OH] | <--> | ARG207 [O] | 2.72 |  |  |  |  |
| ARG231 [NH1] | <--> | GLY239 [O] | 2.90 |  |  |  |  |
| GLN232 [NE2] | <--> | ILE241 [O] | 2.82 |  |  |  |  |
| GLY238 [N] | <--> | TYR228 [O] | 2.82 |  |  |  |  |
| GLY239 [O] | <--> | ARG231 [NH1] | 2.93 |  |  |  |  |
| ILE241 [O] | <--> | GLN232 [NE2] | 2.78 |  |  |  |  |
|  |  |  |  |  |  |  |  |
| **A/C interface interactions** | | | | | | | |
| **Hydrogen Bonds** | | |  | **Salt Bridges** | | | |
| **Chain A** |  | **Chain C** | **Distance (Å)** | **Chain A** |  | **Chain C** | **Distance (Å)** |
| ARG64 [NH1] | <--> | ASP105 [OD2] | 2.87 | ARG64 [NH1] | <--> | ASP101 [OD1] | 3.89 |
| ARG64 [NH2] | <--> | ASP105 [OD1] | 2.90 | ARG64 [NH1] | <--> | ASP105 [OD2] | 2.87 |
| PHE96 [N] | <--> | GLU170 [OE1] | 2.94 | ASP101 [OD2] | <--> | ARG64 [NH1] | 3.36 |
| LEU99 [O] | <--> | TYR116 [OH] | 3.08 | ASP105 [OD2] | <--> | ARG64 [NH1] | 2.89 |
| LEU99 [O] | <--> | LYS120 [NZ] | 2.80 |  |  |  |  |
| ASP101 [OD1] | <--> | TYR116 [OH] | 3.06 |  |  |  |  |
| ASP105 [OD1] | <--> | ARG64 [NH2] | 2.95 |  |  |  |  |
| ASP105 [OD2] | <--> | ARG64 [NH1] | 2.89 |  |  |  |  |
| ASP113 [OD1] | <--> | TRP104 [NE1] | 3.28 |  |  |  |  |
| TYR116 [OH] | <--> | LEU99 [O] | 3.24 |  |  |  |  |
| TYR116 [OH] | <--> | ASP101 [OD1] | 2.80 |  |  |  |  |
| LYS120 [NZ] | <--> | LEU99 [O] | 2.84 |  |  |  |  |
| ILE144 [O] | <--> | LYS165 [NZ] | 3.03 |  |  |  |  |
| MET145 [O] | <--> | ARG231 [NE] | 3.04 |  |  |  |  |
| MET145 [O] | <--> | ARG231 [NH2] | 2.93 |  |  |  |  |
| ARG148 [NE] | <--> | LEU169 [O] | 3.19 |  |  |  |  |
| GLY149 [N] | <--> | GLU170 [OE2] | 2.83 |  |  |  |  |
| LYS165 [NZ] | <--> | ILE144 [O] | 3.24 |  |  |  |  |
| GLU170 [OE1] | <--> | PHE96 [N] | 2.93 |  |  |  |  |
| GLU170 [OE2] | <--> | GLY149 [N] | 2.75 |  |  |  |  |
| ARG231 [NE] | <--> | MET145 [O] | 2.99 |  |  |  |  |
| ARG231 [NH2] | <--> | MET145 [O] | 2.84 |  |  |  |  |

**Supplementary Table 4** Tetramer interface interactions for *Ab*SDR

| **PDB: 6UUT A/B interface interactions** | | | | | | | |
| --- | --- | --- | --- | --- | --- | --- | --- |
| **Hydrogen Bonds** | | | | **Salt bridges** | | | |
| **Chain A** |  | **Chain B** | **Distance (Å)** | **Chain A** |  | **Chain B** | **Distance (Å)** |
| SER112 [OG] | <--> | SER331 [OG] | 2.77 | GLU113 [OE2] | <--> | ARG335 [NH1] | 3.94 |
| SER112 [OG] | <--> | SER331 [OG] | 2.77 | LYS149 [NZ] | <--> | ASP338 [OD2] | 2.60 |
| SER112 [OG] | <--> | GLU334 [OE1] | 2.64 | ARG335 [NH1] | <--> | GLU113 [OE2] | 3.35 |
| TYR119 [OH] | <--> | MET317 [O] | 2.49 | ASP338 [OD2] | <--> | LYS149 [NZ] | 2.69 |
| ASN123 [ND2] | <--> | LEU314 [O] | 2.74 |  |  |  |  |
| ARG127 [NH2] | <--> | ASN316 [OD1] | 2.88 |  |  |  |  |
| LYS149 [NZ] | <--> | ASP338 [OD2] | 2.60 |  |  |  |  |
| SER163 [OG] | <--> | ASN364 [N] | 2.95 |  |  |  |  |
| SER163 [OG] | <--> | ASN364 [O] | 3.26 |  |  |  |  |
| LYS166 [NZ] | <--> | ALA362 [O] | 2.99 |  |  |  |  |
| LYS166 [NZ] | <--> | GLY363 [O] | 2.97 |  |  |  |  |
| GLU167 [OE1] | <--> | GLY366 [N] | 2.84 |  |  |  |  |
| GLU167 [OE2] | <--> | LEU314 [N] | 2.65 |  |  |  |  |
| LEU314 [N] | <--> | GLU167 [OE2] | 2.66 |  |  |  |  |
| LEU314 [O] | <--> | ASN123 [ND2] | 2.85 |  |  |  |  |
| ASN316 [OD1] | <--> | ARG127 [NH2] | 2.71 |  |  |  |  |
| MET317 [O] | <--> | TYR119 [OH] | 2.59 |  |  |  |  |
| SER331 [OG] | <--> | SER112 [OG] | 2.63 |  |  |  |  |
| SER331 [OG] | <--> | SER112 [OG] | 2.63 |  |  |  |  |
| GLU334 [OE1] | <--> | SER112 [OG] | 2.49 |  |  |  |  |
| ASP338 [OD2] | <--> | LYS149 [NZ] | 2.69 |  |  |  |  |
| ALA362 [O] | <--> | LYS166 [NZ] | 3.12 |  |  |  |  |
| GLY363 [O] | <--> | LYS166 [NZ] | 2.97 |  |  |  |  |
| ASN364 [N] | <--> | SER163 [OG] | 2.92 |  |  |  |  |
| ASN364 [O] | <--> | SER163 [OG] | 3.26 |  |  |  |  |
| GLY366 [N] | <--> | GLU167 [OE1] | 2.81 |  |  |  |  |
| ARG454 [NH1] | <--> | ALA463 [O] | 2.97 |  |  |  |  |
| LEU460 [O] | <--> | GLY462 [N] | 3.34 |  |  |  |  |
| GLY462 [N] | <--> | LEU460 [O] | 3.33 |  |  |  |  |
| ALA463 [N] | <--> | ALA463 [OXT] | 2.89 |  |  |  |  |
| ALA463 [O] | <--> | ARG454 [NH1] | 2.87 |  |  |  |  |
| ALA463 [OXT] | <--> | ALA463 [N] | 2.80 |  |  |  |  |

**Supplementary Table 5** Dimer interface interactions for HMwFabG

|  | **ATCC 17978 (µg.ml^-1^)** | ***ΔHmwFabG* (µg.ml^-1^)** |
| --- | --- | --- |
| **Gentamicin** | 2 | 2 |
| **Streptomycin** | 128 | 128 |
| **Chloramphenicol** | 256 | 128 |
| **Colistin** | 4 | 4 |

**Supplementary Table 6. Antibiotic minimal inhibitory concentrations**
